# COVID-19 and output in Japan

**DOI:** 10.1007/s42973-021-00098-4

**Published:** 2021-09-22

**Authors:** Daisuke Fujii, Taisuke Nakata

**Affiliations:** 1 Faculty of Economics, University of Tokyo, 7-3-1 Hongo, Bunkyo-ku, Tokyo, 113-0033 Japan; 2 RIETI (Research Institute of Economy, Trade and Industry), 1-3-1, Kasumigaseki Chiyoda-ku, Tokyo, 100-8901 Japan

**Keywords:** COVID-19, Japan, SIR model, Epidemiological model, Vaccines, State of emergency, Variants, E17, E70, I18

## Abstract

We build a tractable SIR-macro-model with time-varying parameters and use it to explore various policy questions such as when to lift the state of emergency (SOE). An earlier departure from the SOE results in smaller output loss and more deaths in the short run. However, if the SOE is lifted too early, the number of new cases will surge and another SOE may need to be issued in the future, possibly resulting in both larger output loss and more deaths. That is, the tradeoff between output and infection that exists in the short run does not necessarily exist in the long run. Our model-based analysis—updated weekly since January 2021, frequently reported by media, and presented to policymakers on many occasions—has played a unique role in the policy response to the COVID-19 crisis in Japan.

## Introduction

**Fig. 1:**
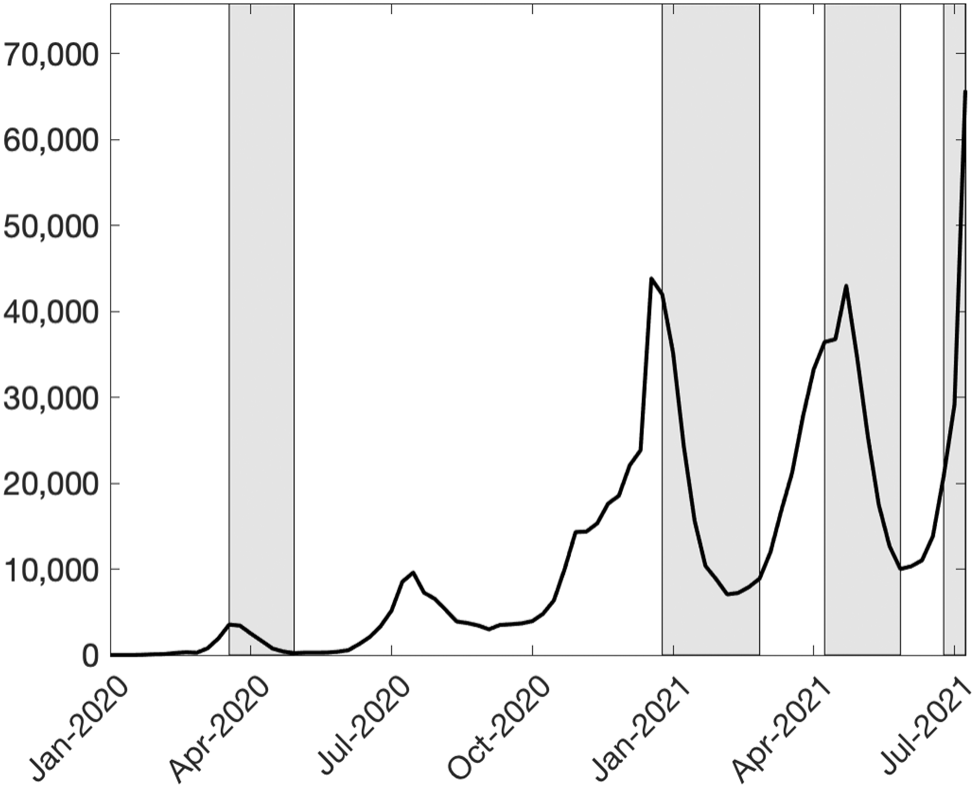
The weekly number of new COVID-19 cases in Japan. Shaded regions indicate the periods of SOE in Tokyo. Source: Ministry of Health, Labor, and Welfare (2020). As of August 8th

Since the outbreak of COVID-19 pandemic in early 2020, many countries have implemented non-pharmaceutical interventions (NPIs) such as stay-at-home orders and city-wide lockdowns to control the spread of coronavirus. Japan is no exception. As shown in Fig. 1, Japan has experienced four epidemic waves, and is in the middle of the fifth wave as of early August in 2021. To abate the spread of infection, the Japanese government has declared the state of emergency (SOE) four times: April 7th–May 25th in 2020 (all prefectures), January 8th–March 21st in 2021 (11 prefectures), April 25th–June 20th in 2021 (10 prefectures), and July 12th–August 31st (6 prefectures). Compared to strict lockdowns employed in many countries, social and economic restrictions of SOE are weaker (Watanabe & Yabu, 2021). Nonetheless, they had a significant impact on economy and seem to have been effective in containing the spread of infection. These experiences sparked social debate on how much we should suppress social and economic activities to control infection.

We build a tractable SIR-macro-model with time-varying parameters and quantify the relationship between the spread of COVID-19 and output in Japan. We then apply the framework to explore various policy questions such as when to lift the state of emergency (SOE) and how to accelerate economic activity. An earlier departure from the SOE results in smaller output loss and more deaths in the short run. However, if the SOE is lifted too early, the number of new cases will surge and another SOE may need to be issued in the future, resulting in more deaths and larger output loss. That is, the tradeoff between output and infection that exists in the short run does not necessarily exist in the long run.

We also investigate the consequences of the spread of more contagious coronavirus variants and faster vaccine rollout. A faster increase in the ratio of a more transmissive variant, be it the Alpha or Delta variant, would lead to a faster increase in the number of new cases, necessitating an earlier invocation of the SOE and larger economic loss. A faster vaccine rollout is associated with smaller economic loss and deaths. If we compare 0.6 M/day and 1.2 M/day, the difference in economic loss is 1.8 trillion yen and that of cumulative deaths is around 400. These numbers are only for Tokyo; they are larger at the national level.

Finally, we extend our framework to a multi-group SIR model to incorporate age heterogeneity, and show simple parameter adjustment of the single-group SIR model allows it to replicate the aggregate dynamics of the multi-group SIR model. More specifically, when vaccines are distributed to elderlies before non-elderlies, an AR(1) adjustment on the transmission rate and a declining path of a mortality rate—that depends on the rate of vaccine rollout—can generate aggregate dynamics that are quantitatively similar to those of a multi-group SIR model with age heterogeneity. Because of data limitation as well as the practical need for frequent assessments of the outlook, we argue that our single-group SIR-macro-model is a viable tool for real-time policy analyses.

The purpose of our project is to provide policy-oriented research in a timely manner. Given unpredictable nature of the evolution of COVID-19, policymakers and the public need to frequently reassess their policies and behaviors in coming weeks and months. To provide a timely analysis on a wide range of policy questions, we deliberately made the baseline model as simple as possible, so as to be able to flexibly accommodate many pragmatic extensions. In particular, our model abstracts from individuals’ optimization problem and welfare analysis, unlike most of the SIR-macro models (e.g., Alvarez et al., 2020; Eichenbaum et al., 2021; Farboodi et al., 2020; Kaplan et al., 2020). As discussed in Sect. 2, we have updated our model-based projections every week since mid-January 2021 and have conducted a diverse set of policy analyses using our model. Our analyses have been presented to the Cabinet Office, Prime Minister’s Office, Advisory Board of Ministry of Health, Labour and Welfare (henceforth, MHLW-AB) (2020), and key policymakers on many occasions. Our analyses have also been conveyed to the Japanese public through a number of newspaper articles and TV news.

In terms of the theoretical framework, our model resembles that of Acemoglu et al. (2021) They also construct a multi-group SIR model without individual’s optimization problem and trace the tradeoff curve between economic loss and deaths from infection. Their focus is to study the optimal targeted lockdown policies which shift the tradeoff curve. Unlike their model, we consider time-varying parameters, and fit past data to obtain reasonable estimates of parameters for future projections. Also, we allow realistic vaccine rollout to different age groups and examine its effect on the reduction in mortality.

Our analysis on the second SOE in Tokyo complements that of Kubota (2021) based on a SIR-macro-model which embeds economic agents’ optimizations. His model can decompose the effects of government’s containment policies and individuals’ behaviors, and translate the degree of soft lockdown to a consumption tax. His results on the SOE in Tokyo are quantitatively and qualitatively similar to our results on the same issue. Our work is different from his work in a number of ways, including, but not limited to, the model, the estimation method, and the range of policy experiments considered.

This paper is organized as follows. Section 2 elaborates how our analyses contributed to the policy discussions on COVID-19 in Japan. Section 3 describes our model and data. Section 4 illustrates conditional projections of COVID-19 and the relationship between economy and infection. Section 5 presents various applications of our framework such as the examination of an exit strategy of the SOE and different vaccine rollout scenarios. Section 6 considers age heterogeneity by extending our model to a multi-group SIR and describes the differences from a single-group SIR model. Section 7 concludes. Unlike a standard academic paper, we describe the history of our real-time policy analyses in what follows. Retrospectively, some of the results are outdated since our specifications have been constantly evolving to reflect fast-moving situation of COVID-19 such as the spread of mutant strains. We believe that it is worthwhile to record the entire history of our analyses including how the model specifications have evolved. For each projection or application, we specify when it was done to clarify the timeline.

## The role of our analysis in Japanese COVID-19 policies

Our research is unique in the sense that the analyses based on our model have acted as a key input to policymakers and the public in Japan. As described in Inaba (2020), prior to the COVID-19 crisis, the Japanese government had invested little in research of mathematical epidemiological modeling. As a result, a sufficient amount of model-based epidemiological analyses were not available to guide key policy discussions in real time.^1^

As of January 2021, when the first draft of this paper was written and our weekly update of the economic and COVID-19 outlook began, Japanese policymakers and public were not regularly presented with any medium-term and long-term outlook of COVID-19 by epidemiologists. The lack of outlook from experts is a stark contrast with what you see in the UK where a group of epidemiologists regularly provided the government and public with medium-term and long-term outlook of infection.

Against this backdrop, our weekly projections of the economy and COVID-19 garnered immediate attention of policymakers and the public in Japan. From early February to late March, our analysis of the second SOE in Tokyo—described in Sect. 5—was frequently featured by Japanese media. In early February, there was a disagreement among the government and public-health experts as to how much infection should decline before lifting the SOE, with the government seemingly inclined to lift the SOE and stimulate economic activity sooner than public-health experts would find desirable. Our analysis provided economic justification for “being patient” in lifting the SOE. The government ended up with extending the second SOE twice, first from February 7th to March 7th and second, from March 7th to March 21st.

Our analysis also contributed to the policy debate on whether to hold the Tokyo Olympics and, if so, how many spectators should be allowed in Olympics venues. The public debate regarding the Olympics began to intensify in April, with the majority of public expressing skepticism towards the feasibility of safely holding the Olympics. Yet, there was no quantitative analysis regarding how the Tokyo Olympics may affect the spread of the disease. Our May 21st analysis based on a variation of the model presented in this paper was the first quantitative analysis to shed light on this important policy question (Fujii & Nakata, 2021; Fujii et al., 2021). Our analysis concluded that the marginal effects of welcoming about 100,000 foreign visitors (athletes, media staffs, and organizers) would be very limited given the large number of population in Tokyo (14 million) and that if the Tokyo Olympics were to have significant effects on infection in Tokyo, that would come from how Tokyo residents behave during the Olympics. Our analysis played a crucial role in directing the attention of policymakers and the public to where they should focus to minimize the risk of Olympics-induced infection.^2^

Our analysis was the first to provide Japanese policymakers and the public with the outlook of COVID-19 infection that incorporates the effects of rising shares of Alpha and Delta variants. As discussed in Sect. 5.2, we began to incorporate the effects of the rising share of the Alpha variant in generating the outlook on March 30th, 2021. Our outlook—the first in Japan to consider the effects of the Alpha variant—was shared with key policymakers, including, but not limited to, Tokyo Governor.^3^ We began to incorporate the rising share of the Delta variant in our outlook on May 21st, 2021, providing the Japanese public early warning of what’s to come in the near future.^4^

Since April, we have received numerous requests from various parties, including the Cabinet Office, Prime Minister’s Office, and members of the Subcommittee on Novel Coronavirus Disease Control and MHLW-AB. Table 1 summarizes the timing of our analyses and key policy briefings for the first half of 2021.

**Table 1:** Timeline of key policy briefings and events in the first half of 2021

Date	Events
January 22nd	Began the weekly update of outlook
February 10th	Subcommittee on Novel Coronavirus Disease Control
March 30th	Released “How the Alpha variant affects the outlook in Tokyo”
April 8th	Tokyo COVID-19 monitoring meeting
May 8th	Prime Minister’s Office
May 21st	Released “The Effects of the Tokyo Olympics on COVID-19: A Quantitative Analysis”
May 21st	Released “How the Delta variant affects the outlook in Tokyo”
May 28th	Olympics Committee Experts Round-table
June 2nd	37th MHLW Advisory Board meeting
June 16th	39th MHLW Advisory Board meeting
June 17th	Released “The Effects of the Tokyo Olympics on COVID-19: The Role of Spectators”
June 18th	Olympics Committee Experts Round-table
June 20th	Prime Minister’s Office
June 30th	41st MHLW Advisory Board meeting
June 30th	Council of Ministers

## Theoretical framework

This section describes our baseline model and elaborates how to back out time-varying parameters. As mentioned above, our model has developed over time to respond to fast-moving situations of infection. Models in the following sections are slightly different each other. Table 2 summarizes various versions of our model.

**Table 2:** Development of model specifications

	Sections 3 and 4	Section 5.1	Section 5.2	Section 5.3
Date published	January 2021	January 2021	March 2021	June 2021
Adjustment of the projection path of mortality rate	No	Yes	Yes	Yes
Alpha variant	No	No	Yes	Yes
Delta variant	No	No	No	Yes
ICU patients	No	No	No	Yes
Adjustment of the projection path of transmission rate	No	No	No	Yes

“Adjustment of the projection path of mortality rate” means whether we take into account the decline in mortality rate due to the composition effect of the infected. Because of the prioritized vaccine rollout for the elderly, the share of older individuals whose mortality rate is much higher is expected to shrink among the infected. “Alpha variant” and “Delta variant” means that an increase of the transmission rate due to the spread of the variants is incorporated using a logistic function. “ICU patients” means that the number of severe cases is considered. “Adjustment of the projection path of transmission rate” means that the transmission rate is adjusted by a positive AR(1) shock process to generate comparable results with a multi-group SIR model

### Model

Our model is formulated in a discrete time with each period interpreted as a week. It consists of two parts: the epidemiological and economic part. The epidemiological part is given by the following SIRD model:

1 $$\begin{aligned} S_{t+1}-S_{t}=-N_{t}-V_{t}, \end{aligned}$$

2 $$\begin{aligned} I_{t+1}-I_{t}=N_{t}-N_{t}^{IR}-N_{t}^{ID}, \end{aligned}$$

3 $$\begin{aligned} R_{t+1}-R_{t}=N_{t}^{IR}+V_{t}, \end{aligned}$$

4 $$\begin{aligned} D_{t+1}-D_{t}=N_{t}^{ID}, \end{aligned}$$

5 $$\begin{aligned} N_{t}^{IR}=\gamma _{t}I_{t}, \end{aligned}$$

6 $$\begin{aligned} N_{t}^{ID}=\delta _{t}I_{t}. \end{aligned}$$

$S_{t}$, $I_{t}$, and $R_{t}$ denote the number of susceptible, infectious, and recovered individuals, respectively. $D_{t}$ denotes the number of cumulative deaths. The total population is denoted by $POP_{0}$. Since we do not consider birth and other sources of deaths, the total population is preserved at any time *t*

$$\begin{aligned} S_{t}+I_{t}+R_{t}+D_{t}=POP_{0}\quad \text { for any }t. \end{aligned}$$

The flow variables $N_{t}$, $N_{t}^{IR}$, and $N_{t}^{ID}$ are the number of newly infected persons, newly recovered persons, and deaths from COVID-19 between time *t* and time $t+1$, respectively. $V_{t}$ is the number of newly vaccinated persons from time *t* to time $t+1$. Time-varying parameters $\gamma _{t}$ and $\delta _{t}$ are Poisson rates for recovery and death from the infected, respectively.^5^

The economic part of our model is given by the following linear production function.

7 $$\begin{aligned} Y_{t}&=(1-\alpha _{t})A_{t}(\{\alpha _{\tau }\}_{\tau =0}^{t-1})(S_{t}+I_{t}+R_{t})\nonumber \\&:=(1-\alpha _{t}){\bar{Y}}_{t}. \end{aligned}$$

$Y_{t}$ is output and depends on (i) the population of alive individuals given by ($S_{t}+I_{t}+R_{t}$)—and (ii) output per person—given by $(1-\alpha _{t})A_{t}(\{\alpha _{\tau }\}_{\tau =0}^{t-1})$. Output per person consists of two components. The first component, $(1-\alpha _{t})$, captures the reduction in output per person due to social-distancing or other measures aimed at reducing the risk of infection. The second component, $A_{t}$, is output per person that would prevail if no person takes measures against the risk of infection at time *t*. The dependence of $A_{t}$ on the history of $\alpha _{\tau }$ is intended to capture possible hysteresis effects of having restrained economic activities in the past. We use ${\bar{Y}}_{t}$ to denote the level of output that would prevail if no one restrained his or her economic activities at time t and refer to it as the *reference level of output*. Appendix 1 describes in detail what we intend to capture by the reference level of output as well as how we construct it.

The epidemiological part of our model is linked to the economic part through the following matching function for newly infected persons.

8 $$\begin{aligned} N_{t}=\frac{{\tilde{\beta }}_{t}}{POP_{0}}I_{t}S_{t}, \end{aligned}$$

where

9 $$\begin{aligned} {\tilde{\beta }}_{t}=\beta _{t}(1-h\alpha _{t})^{k}. \end{aligned}$$

${\tilde{\beta }}_{t}$ denotes the transmission rate. $\beta _{t}$ denotes the “output-adjusted” or “raw” transmission rate that would prevail in the absence of any decline in economic activity. $\beta _{t}$ falls if people take actions that reduce the infection risk but do not directly affect their economic activities. For example, $\beta _{t}$ falls if people wear masks or wash their hands when they return home. $\beta _{t}$ also reflects the intrinsic transmission rate of COVID-19. If a coronavirus variant with a higher infectious capacity spreads, it will appear as a higher value of $\beta _{t}$.

The term $(1-h\alpha _{t})^{k}$ captures the effect of a decline in economic activity on the transmission rate. We assume quadratic matching of the susceptible and the infected and assume $k=2$.^6^ It is helpful to think of $\left( 1-h\alpha _{t}\right)$ as a proxy of people’s mobility. While some mobility is necessary for households to consume and businesses to produce goods and services, it leads to interactions between susceptible and infectious persons and thus helps spread the disease. The elasticity of economic loss on mobility is denoted by *h*. A high value of *h* means that the transmission rate can be reduced a lot without reducing output that much. The value of *h* captures, among others, teleworkability of office work, abilities of restaurants to raise revenues through take-out services, or consumers’ willingness to switch from movie theaters to online streaming services.

Our model is not micro-founded, unlike many macro-epidemiological models recently developed in the economics profession. The advantage of our modeling approach lies in the absence of tight cross-equation restrictions, which enables us to fit the past data well, solve the model quickly, and conduct a broad set of policy experiments in a short period of time. The disadvantage of our approach is that our analysis is subject to the Lucas critique. We judge that the advantages outweigh the disadvantage in our work because the main goal of our project is to provide with policymakers and the public future outlook and the effects of various policies in a timely manner.^7^

### Data and identification of unobserved variables and time-varying parameters

We use data on $N_{t}$, $N_{t}^{ID}$, and $Y_{t}$ to recover the paths of the model variables and time-varying parameters. $N_{t}$ and $N_{t}^{ID}$ are the number of new positive PCR test cases and the number of deaths due to COVID-19, respectively, from the Ministry of Health, Labour and Welfare (2020) in Japan. $Y_{t}$ is based on monthly estimates of real GDP computed by the Japan Center for Economic Research.^8^ We assume the following initial conditions: $S_{0}=125.7\,M$, $I_{0}=1$, $R_{0}=0$, and $D_{0}=0$. Throughout the analysis, we set $\gamma _{t}=7/18$, which is the value used in Eichenbaum et al. (2021). This implies that the average duration for an infected individual to recover or die is 18 days. The path of vaccinated population $V_{t}$ is computed as follows. Let $E_{1}$ be the share of population with the first dose of vaccine who can move from *S* to *R*. Define $E_{2}$ analogously for the second shot of vaccine. With $V_{1,t}$ and $V_{2,t}$ be the number of first and second shots of vaccines, respectively, we have

$$\begin{aligned} V_{t}=E_{1}V_{t,1}+\left( E_{2}-E_{1}\right) V_{2,t}. \end{aligned}$$

The time-series data of $V_{1,t}$ and $V_{2,t}$ are retrieved from the MHLW website. We assume Pfizer vaccines are used and set $E_{1}=0.625$ and $E_{2}=0.895$ based on the UK’s SPI-M-O Summary on March 31st, 2021.^9^

Given $S_{0}$ and the paths of $V_{t}$ and $N_{t}$, we can recover the path of $S_{t}$ using Eq. (1). Given $I_{0}$ and the paths of $N_{t}^{ID}$ and $\gamma _{t}$, we can recover the path of $I_{t}$ using Eq. (2). Given $R_{0}$ and the path of $I_{t}$, we can recover the path of $R_{t}$ using Eq. (3). Given $D_{0}$ and the path of $N_{t}^{ID}$, we can recover the path of $D_{t}$ using Eq. (4).^10^Once we recover the path of $D_{t}$, we can recover the path of the death rate ($\delta _{t}$) using Eq. (6). Once we recover the paths of $I_{t}$ and $S_{t}$, we can use Eq. (8) to recover the path of ${\tilde{\beta }}_{t}$.

We make an assumption about the path of ${\bar{Y}}_{t}$, which is described in detail in Appendix 1. Our assumed path grows very slowly over time and is essentially flat. Using the assumed path of ${\bar{Y}}_{t}$, we can recover the path of $\alpha _{t}$ using Eq. (7). Because of the essential flatness of ${\bar{Y}}_{t}$, fluctuation of $\alpha _{t}$ largely inherits that of $Y_{t}$.

**Fig. 2:**
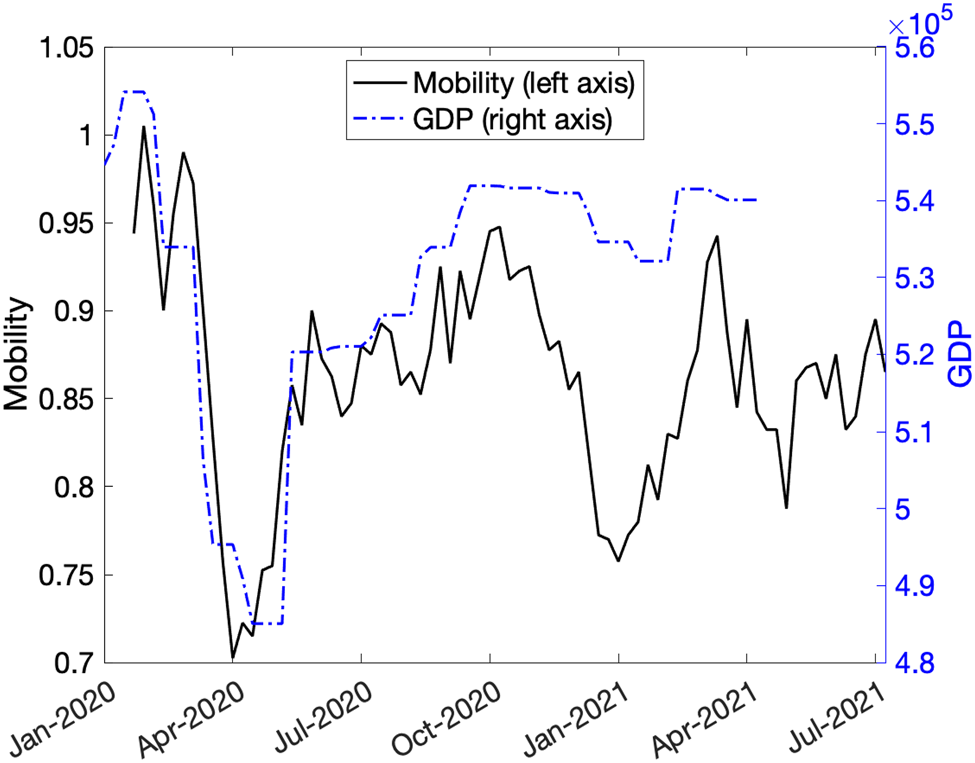
Mobility and output. Source: Japan Center for Economic Research and Google. As of August 8th, 2021

We obtain an estimate of *h* by regressing the Google mobility index ($M_{t}$) on $\alpha _{t}$.^11^ As shown in Fig. 2, the correlation between $M_{t}$ and $Y_{t}$ is high, and thus the correlation between $M_{t}$ and $\alpha _{t}$ is also high because ${\bar{Y}}_{t}$ is essentially flat. We run the following simple regression

$$\begin{aligned} M_{t}=h_{0}+h_{1}\alpha _{t}+\epsilon _{t}, \end{aligned}$$

where $\epsilon _{t}$ is the residual. Using the data up April 2021, the estimated values are ${\hat{h}}_{0}=0.923$ and ${\hat{h}}_{1}=-1.41$. We then compute the estimate of *h* as follows

$$\begin{aligned} {\hat{h}}=\frac{{\hat{h}}_{1}}{{\hat{h}}_{0}}=-1.52 \end{aligned}$$

The reason why we divide the slope coefficient by the intercept is for normalization. In the above regression specification, the intercept $h_{0}$ is the mobility when there is no economic loss. We normalize the elasticity $h_{1}$ by $h_{0}$ to obtain the estimate of *h* consistent with Eq. (9).

**Fig. 3:**
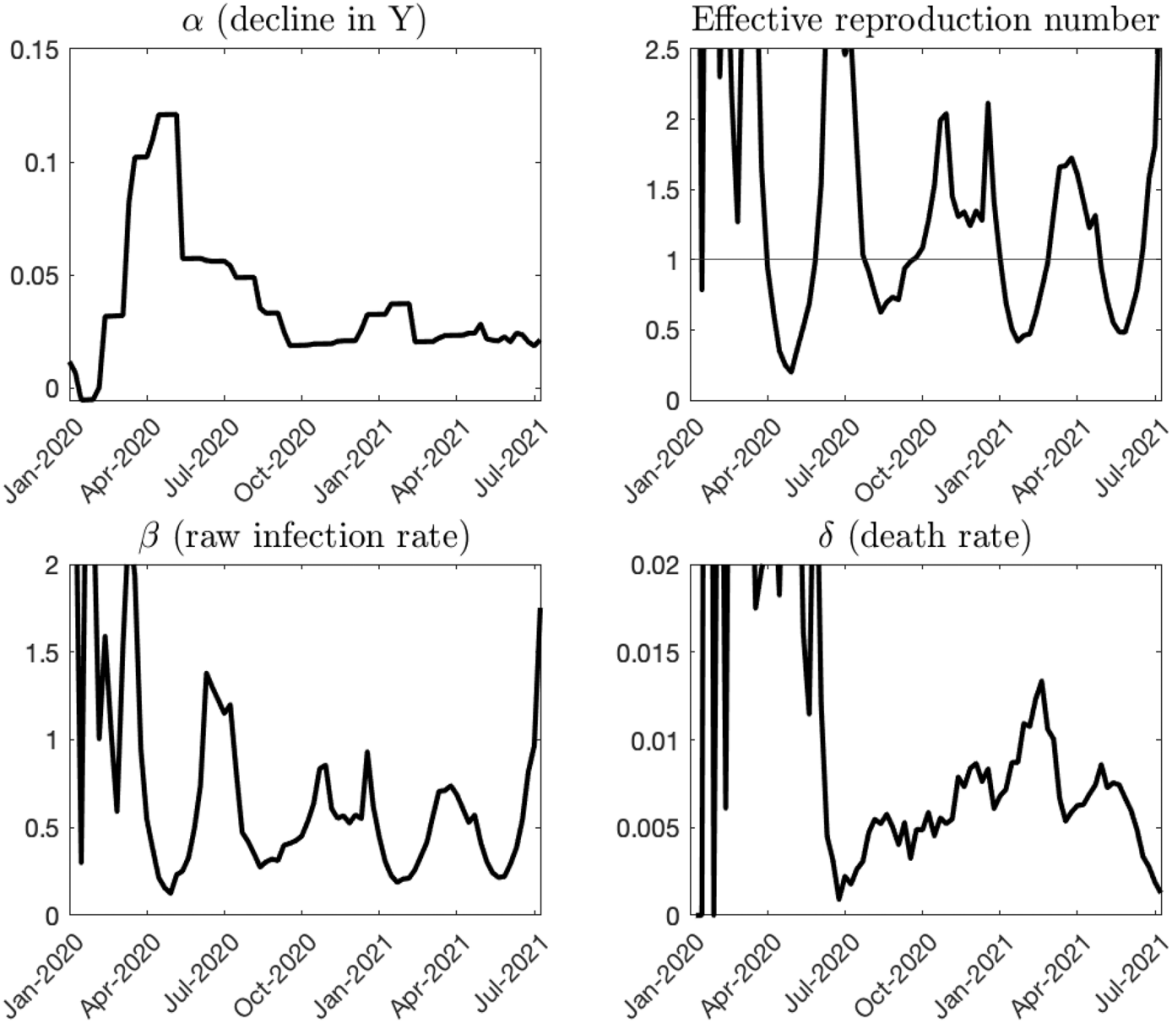
History of time-varying parameters. Source: Authors’ calculation. As of August 8th, 2021

**Fig. 4:**
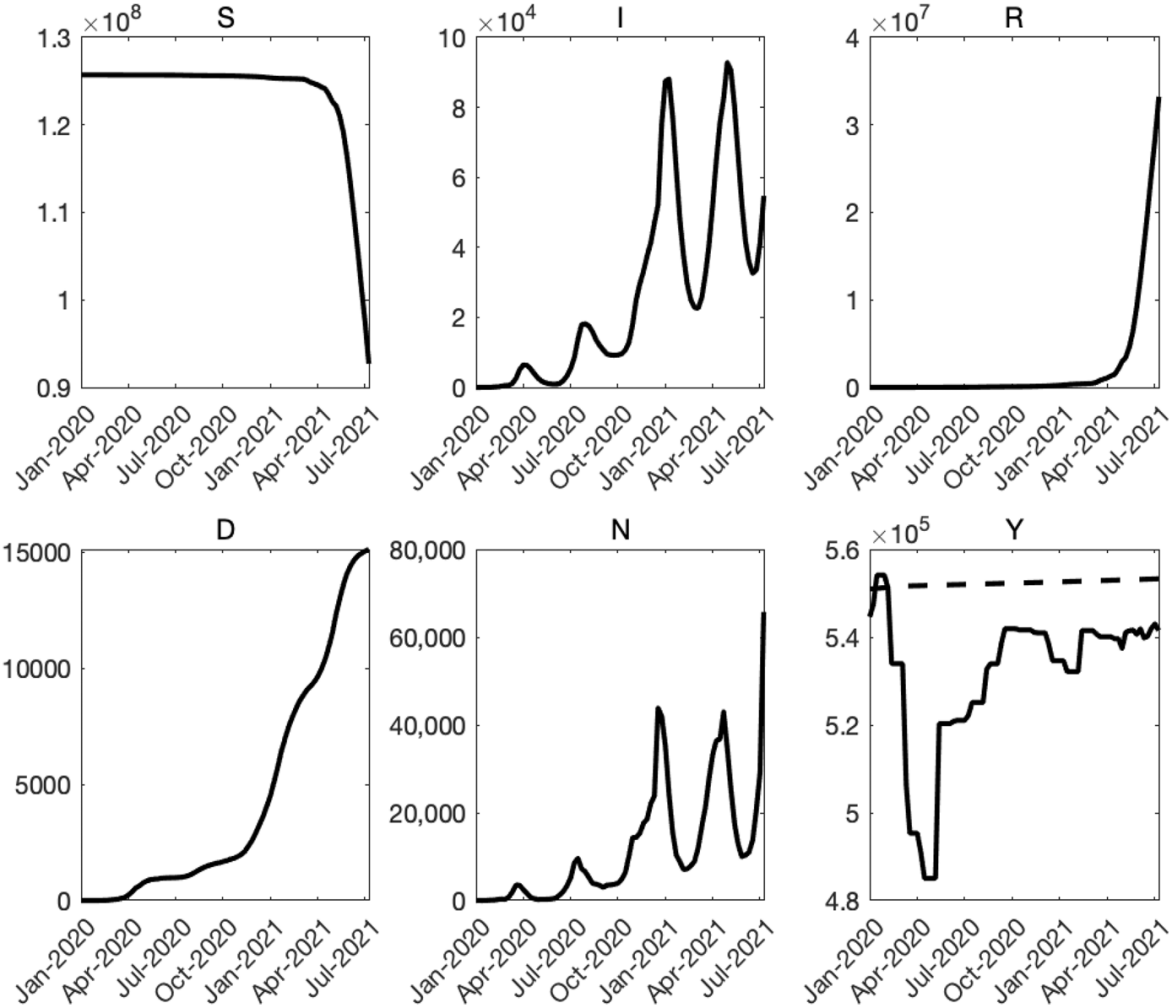
History of COVID-19 and output. Note: In the bottom-right panel, the dashed line shows the reference level of output. Source: Authors’ calculation, Japan Center for Economic Research, Ministry of Health, Labor, and Welfare (2020). As of August 8th

Given the path of $\alpha _{t}$ and the estimated *h*, we can recover the path of $\beta _{t}$ using Eq. (9). Figures 3 and 4 show the identified paths of time-varying parameters and model variables, respectively.

## Conditional projections of COVID-19 (January 2021)

Analysis in this section was conducted in January, 2021.

We use our model to compute projections of COVID-19 conditional on various paths of output. In computing these conditional projections, we make the following assumptions regarding the evolution of time-varying parameters.

We assume that the death rate ($\delta _{t}$) and the output-adjusted transmission rate ($\beta _{t}$) will be constant at their average values over the most recent 5 months. We picked the 5-month horizon because it minimized the root mean squared error (RMSE) of the projected path of new cases ($N_{t}$) in the past data as of January 2021. The time horizon which minimizes the RMSE of the past data changes over time. Since March 2021, we employ a 4-month horizon to compute the average values of $\beta$ and $\delta$ for projection.

We assume that the vaccine distribution begins in the first week of March 2021.^12^ The number of vaccine shots administered will increase from zero in the last week of February 2021 to 4 M in the final week of May 2021. Thereafter, 4 M vaccine shots will be administered per week over the projection horizon.

We assume that a person receives the second vaccine shot 28 days after the first vaccine shot. For simplicity, we assume that a person remains susceptible between the first and second shots and that 80% of persons who have received two vaccine shots will obtain full immunity. With these simplifying assumptions, we obtain our baseline projection of $V_{t}$ that increases from zero in the last week of March 2021 to 1.6 M ($\approx 0.8 \times 4$ M/2) in the final week of June 2021. Thereafter, $V_{t}$ will remain 1.6 M throughout our projection horizon.

We condition our projection on various simple paths of $\alpha _{t}$. In particular, we consider a set of paths whose initial value (the first week in the projection) is positive, declines linearly to zero in the first 6 months, and remains at zero thereafter. This pattern is intended to parsimoniously capture the pattern of $\alpha$ in the aftermath of the national emergency declaration in early April 2020.

**Fig. 5:**
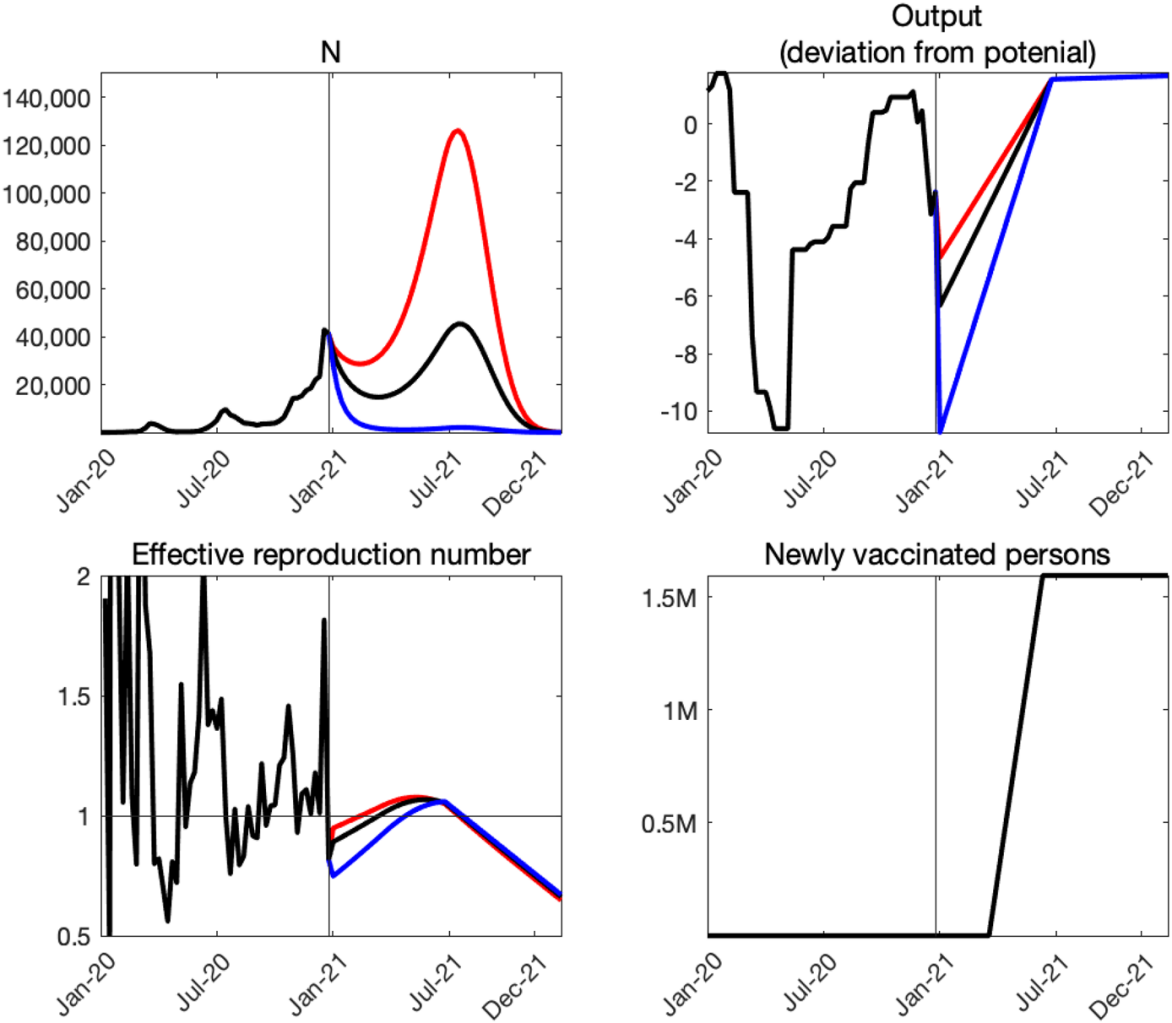
Conditional projections of COVID-19

Figure 5 shows our projection of COVID-19 conditional on three alternative paths $\alpha _{t}$. The average output loss over the next 12 months associated with these paths are $1.5\%$, $2\%$, and $3\%$—shown by the red, black, and blue lines, respectively. According to the figure, suppressing the economic activity to a level in line with that observed in the aftermath of the national emergency declaration of April 2020 does a very good job in containing the spread of COVID-19, as can be seen in the blue lines. The economic decline that is half of this most severe case—shown by the red lines—would initially lead to a small transitory decline in the number of new infections, followed by another surge which peaks around July 2021. Under the intermediate case—shown by the black lines—the number of new infections declines by about half in the first 3 months. As the economic activity picks up again, the number of new infections would increase and peaks in July 2021 at a level similar to the first 2 weeks of January 2021. Thereafter, the number of new infections declines slowly as the vaccine distribution progresses.

### Relationship between COVID-19 and the economy

#### Baseline case

**Fig. 6:**
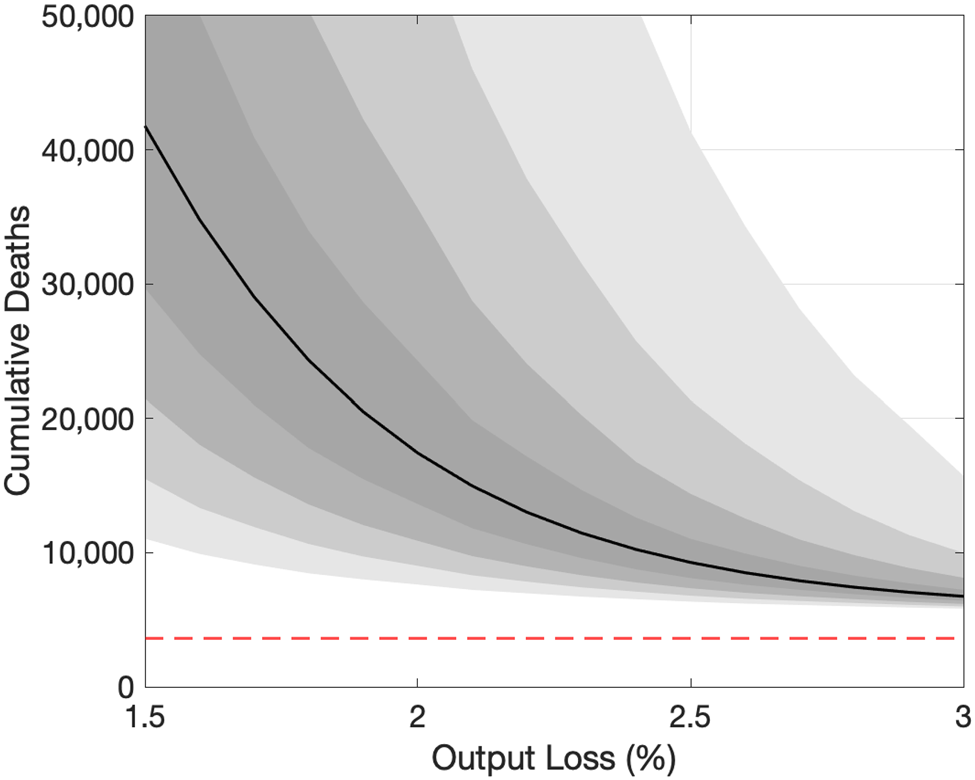
Projected relationship between COVID-19 and output . Note: The vertical axis shows the number of cumulative deaths by the end of the next 12 months. The horizontal dashed line indicates the total number of COVID-19 deaths during 2020. Source: Authors’ calculation

The solid black line in Fig. 6 shows the set of pairs of cumulative deaths by the end of the next 12 months (January 2022) and the average output loss over the next 12 months associated with various paths of $\alpha _{t}$. As a reference, with our imputed $Y_{t}$ for December 2020, the average output loss in 2020 is a touch below 4%. Recent projections for the growth rate of GDP in Japan by IMF, OECD, and World Bank are 2.3%, 2.3%, and 2.5%, respectively. The output loss in 2021 consistent with these growth projections is about 1.6%.^13^

According to the figure, our model predicts 7000, 17,000, and 42,000 deaths by the end of the next 12 months if the average output loss over the next 12 months is $3\%$, $2\%$, and $1.5\%$, respectively. As discussed later, these values are substantially higher than what our model projected just 2 weeks ago, reflecting a sharp spike in the number of new infections a week ago (the week ending in January 10th, 2021). Although we expect the curve to shift down in coming weeks, these numbers indicate just how striking the recent spike was.

Our trade-off curve is concave, reflecting the explosive dynamics inherent in the SIRD model as well as our nonlinear specification of the matching function. One key implication of this concavity is that we can save more lives by reducing output by one unit when the output loss is expected to be small and the disease is out of control than when it is expected to be large and the disease is contained pretty well. In other words, there are *diminishing returns to scale* to reducing output.

Gray areas with varying darkness indicate the degree of uncertainty regarding the relationship between COVID-19 and output. The darkest and the second darkest gray areas indicate 20 and 40% confidence sets, respectively. The second lightest and the lightest gray areas indicated 60 and 80% confidence sets, respectively. These confidence sets are constructed as follows. We compute the standard error of the estimated *h* as well as the standard errors of the average values of the raw transmission rates and deaths rates.^14^. Assuming that they are independently and normally distributed, we draw 40,000 sets of *h*, $\beta$, and $\delta$. For each draw, we compute the trade-off curve. We then look at different percentiles at each level of output loss.

We highlight two features of uncertainty about the trade-off curve. First, uncertainty is higher when the expected output loss is smaller. This feature is driven by the explosive dynamics inherent in the SIRD model: a small difference in the effective reproductive number makes a larger difference in the number of cumulative deaths when the effective reproductive number is higher. Second, uncertainty is high at any level of output loss. Even at the right edge of the figure in which the output loss is 3% and uncertainty appears relatively low, the 80% confidence set ranges from 6000 to 16,000.^15^

#### Sensitivity analysis

**Fig. 7:**
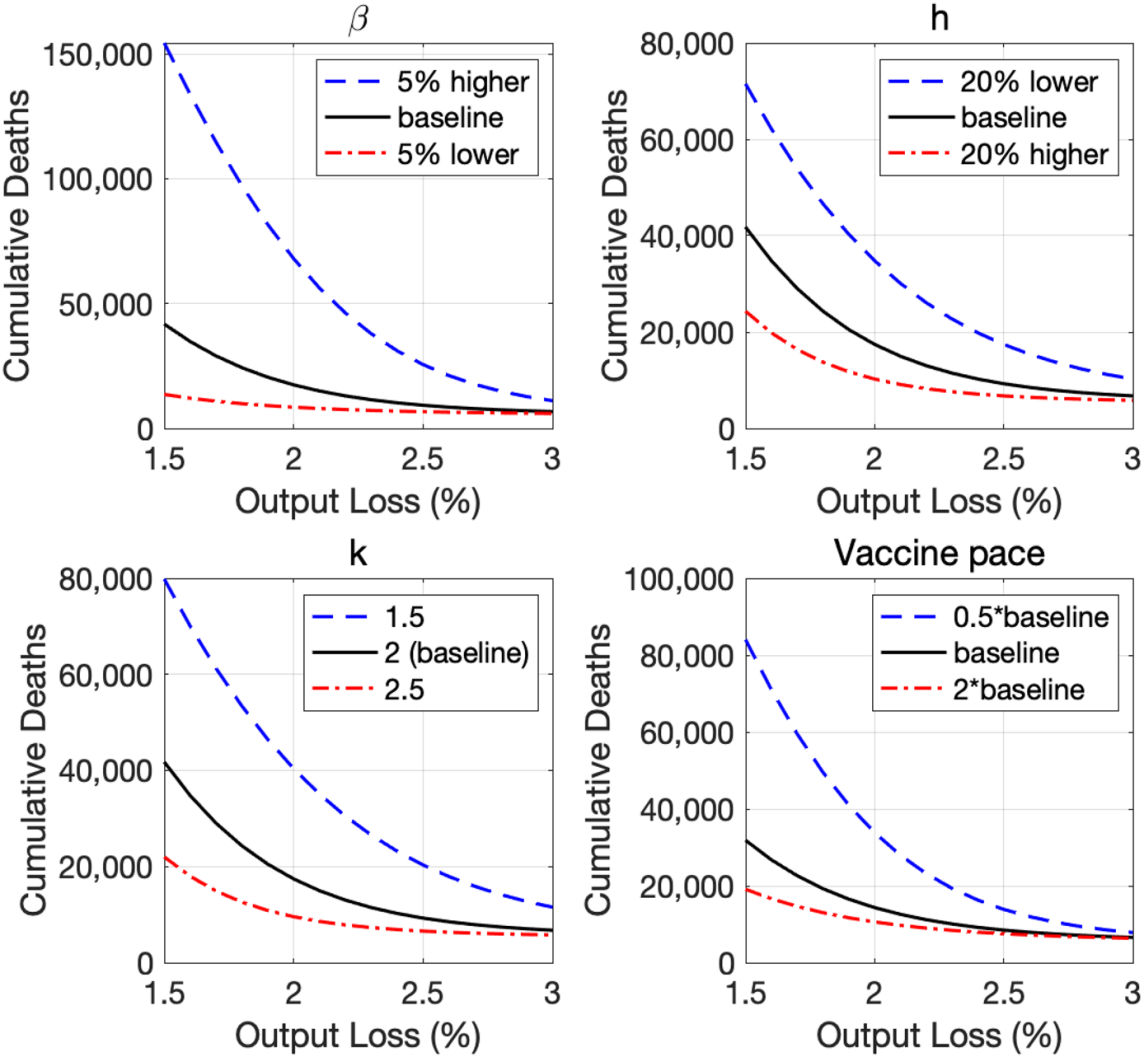
Sensitivity analysis. Note: The vertical axis shows the number of cumulative deaths by the end of the next 12 months

Figure 7 shows how deviations from our baseline specifications affect the relationship between COVID-19 and the economy.

The top-left panel of Fig. 7 shows how the trade-off curve depends on the assumed path of the raw transmission rate. The panel shows that a small increase in the raw transmission rate increases the number of cumulative deaths by tens of thousands if the output loss is expected to be small.

The top-right panel demonstrates the importance of improving teleworkability, promoting flexible work arrangement, or encouraging households and businesses to substitute their economic activities in contact-intensive sectors with those in less contact-intensive sectors.

The bottom-left panel demonstrates how sensitive our projection is to the specification of the matching function for new infections. In the baseline projection, we assume a quadratic function for how $\alpha$ affects the transmission rate—recall $(1-h\alpha _{t})^{2}$ in Eq. (9). The panel considers two alternative functions—$(1-h\alpha _{t})^{1.5}$ and $(1-h\alpha _{t})^{2.5}$.

The bottom-right panel demonstrates the benefit of distributing vaccines at a faster pace. One key feature of the panel is that the benefit of distributing vaccines at a pace faster than in the baseline scenario is larger when the output loss is expected to be smaller. When the output loss is expected to be large, the disease is contained pretty well anyway so that the marginal value of a faster vaccine distribution is smaller.

These analyses demonstrate the importance of policymakers and the public to pursue policies or individually take actions to shift the trade-off curve down. Even though short-run lockdown policies are sometimes necessary to contain the spread of the disease in response to a sharp increase in the number of new infections, they do adversely affect the economy at least in the short run. Better health policies and better individual habits contain the spread of the disease without necessarily reducing output.

#### Evolution of the relationship

**Fig. 8:**
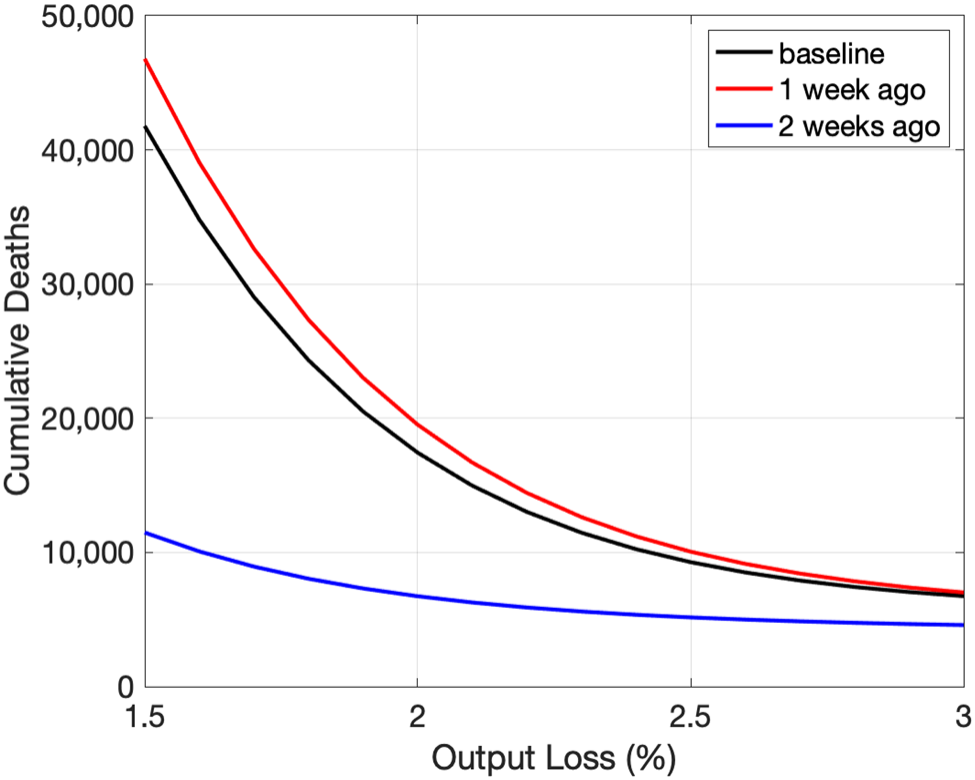
Evolution of the tradeoff curve. Note: The vertical axis shows the number of cumulative deaths by the end of the next 12 months

The projection of COVID-19 depends on the initial conditions as well as the projected paths of the raw transmission rate and the death rate, which in turn depend on the recent realizations of them because we use the average values over the most recent 5 months for projection. As a result, the projected trade-off varies over time. To illustrate this point, Fig. 8 compares the baseline trade-off curve shown earlier (January 17th, 2021) with trade-off curves computed 1 and 2 weeks earlier (January 10th and January 3rd, 2021) using the data available up to that point.

The most recent trade-off curve is a touch below the curve from 1 week ago and substantially above the curve from 2 weeks ago. Because the number of new infections was substantially lower 2 weeks ago than now, the 5-month average values of the raw transmission rate was substantially smaller back then. Accordingly, the number of cumulative deaths was substantially smaller 2 weeks ago than now.

#### Relationship between COVID-19 and output in 2020

**Fig. 9:**
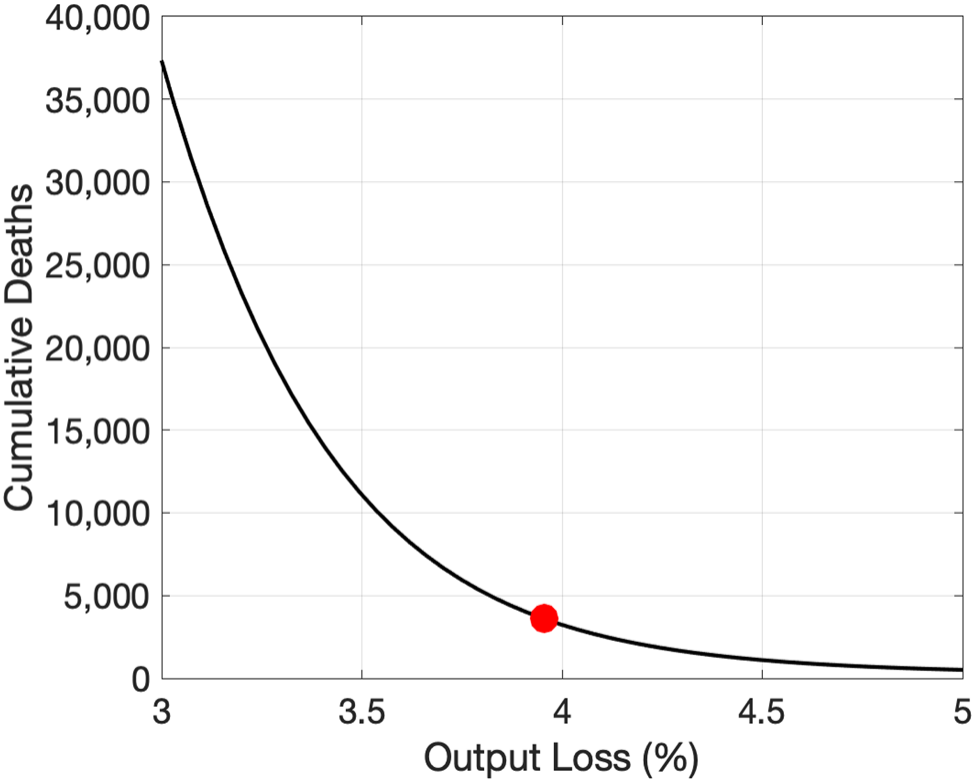
Relationship between COVID-19 and output in 2020. Note: The vertical axis shows the number of cumulative deaths by the end of 2020. The red filled circle indicated the realized pair of death and the average output loss in 2020

Figure 9 shows the relationship between the number of deaths and the average output loss in 2020, based on counterfactual simulations of COVID-19—computed conditional on various counterfactual paths of $\alpha _{t}$. In this exercise, our counterfactual simulation starts from the third week of January 2020 when the first COVID-19 case was reported. We consider counterfactual paths of the economy in which the path of $\alpha _{t}$ is multiplied by a constant for any time *t* from the last week of January 2020 to the last week of December 2020. In this way, we rescale the path of economic loss preserving its shape to generate variations in average output loss. We find that, if the average output loss in 2020 had been $3\%$ and $5\%$, instead of the actual $3.9\%$, the number of deaths in 2020 would have been about 38,000 and 530, respectively, instead of the realized value of 3598.

The slope of the trade-off curve at the realized pair of deaths and the output loss—shown by the red circle—can be seen as capturing the value of statistical life, albeit with many caveats. The implied value of statistical life based on the slope of the curve in this figure is 145 years. In a future work, we plan to apply our methodology to other countries and explore cross-country heterogeneity in the implied value of statistical life based on realized health and economic outcomes in 2020.^16^

### Discussion

There are many factors absent in our analysis but that would be of utmost importance if this type of analysis were to be used to inform the decisions of policymakers and the public. Here, we discuss two such factors—hospital capacity and suicides.

We abstract from a potential increase in the death rate due to hospital congestion. Not only do overcrowded hospitals contribute to an increase in the death rate among COVID-19 patients, they also increase the death rate from other diseases by constraining the supply of medical resources. Taking this consideration into account would increase the overall deaths associated with COVID-19—both direct and indirect—particularly when the output loss is small and the number of COVID-19 cases is large.

We also abstract from a potential increase in the number of suicides associated with prolonged economic distress. According to Chen et al. (2009), the number of suicides per capita is more responsive to the unemployment rate in Japan than in other countries. The average unemployment rate in the first 11 months of 2020 is 2.8%, up from 2.4% in 2019. The number of suicides in the first 11 months of 2020 is 19,225—up from 18,675 in the first 11 months of 2019—reversing the decade-long downward trend for the first time.^17^ Some private-sector analysts expect the unemployment rate to edge up in 2021, which could push up the number of suicides further going forward.

### A real-time assessment of forecasting performance

If a model were to be used as a guidepost for policy, it would be important to be aware of how reliable the model is in explaining the past and predicting the future. Because we assume time-varying parameters, we fit the past data perfectly by construction. To quantify how reliable our model may be in predicting the future, we now examine a real-time forecasting performance of our model.^18^

For each week from the first week of September 2020 to the first week of January 2021, we compute the number of new infections and new COVID-19 deaths over the next week and the next 4 weeks, applying the same forecasting procedure we described earlier and using the data available up to that point. In particular, at each point in time, we re-estimate *h* by regressing the mobility index on $\alpha$ available and compute the projected paths of transmission and death rates from their recent-5-month averages. We evaluate our model’s forecasts conditional on the realized path of $\alpha _{t}$, as our paper focuses on conditional projections of COVID-19.

**Fig. 10:**
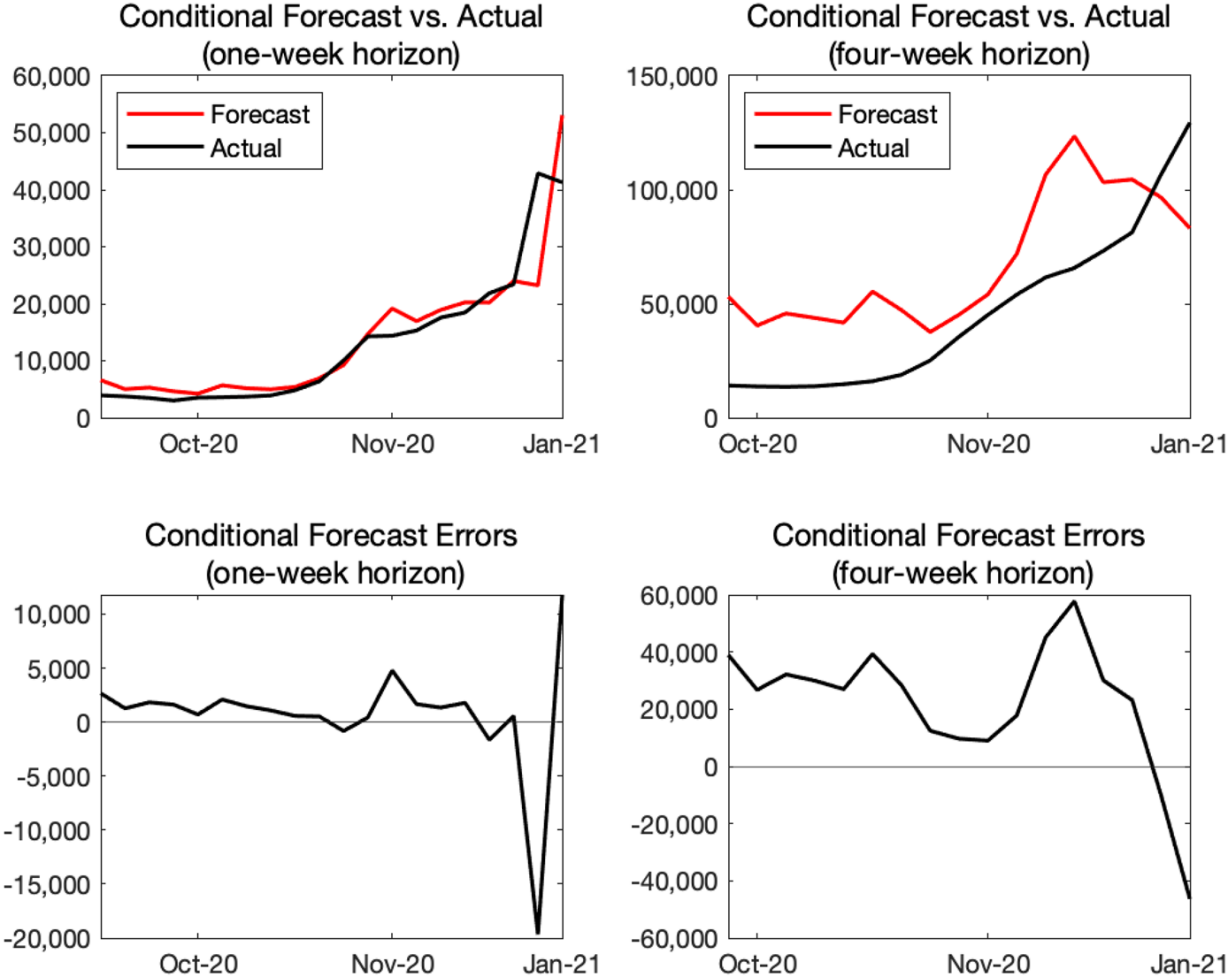
Real-time forecast evaluation: new infections

Our exercise is not fully real-time for two reasons. First, the monthly GDP data produced by the Japan Center for Economic Research is revised historically every month, but we abstract from that historical revision in this exercise. Second, we assume that the weekly GDP data—which is imputed from the monthly GDP data—becomes available after 6 weeks. In practice, the discrepancy between the week in which conditional projections are prepared and the week for which the most recent GDP is available depends on which week of the month the projection is prepared. For example, if one is preparing projections in the final week of December 2020, the most recent monthly GDP is from October 2020 and one needs to impute 7 weeks of GDP. However, if one is preparing projections in the second week of January, the monthly GDP for November 2020 is available and one only needs to impute 5 weeks of GDP. As of February 2021, we used the Google mobility index, which is updated every day with three- or four-day lags, to impute the not-yet-released weekly GDP. More specifically, we invert the regression equation for estimating *h*, and use the estimated coefficients to impute $\alpha _{t}$ given the values of $M_{t}$. Later, we incorporate other highly prompt data such as credit card usage or retail POS data to impute the weekly GDP. See Fujii and Nakata (2021) for more details on how to construct a measure of monthly output in Japan.

Top panels in Fig. 10 show actual and forecasted outcomes for the number of new infections for one- and 4-week horizons, whereas bottom panels show their differences—forecast errors. For the 1-week-ahead projection, our model’s conditional forecast tracks the overall contour reasonably well but often misses actual outcomes by a large amount. For example, our model predicted about 23,000 new cases for the first week of January, 2021 in the final week of December, which is substantially smaller than about 43,000 cases we observed. For the 4-week-ahead projection, our model systematically over-predicted the number of new infections for most of 2020. However, our model’s 4-week forecasts prepared in the first 2 weeks of December 2020 under-predicted the number of new infections.

**Fig. 11:**
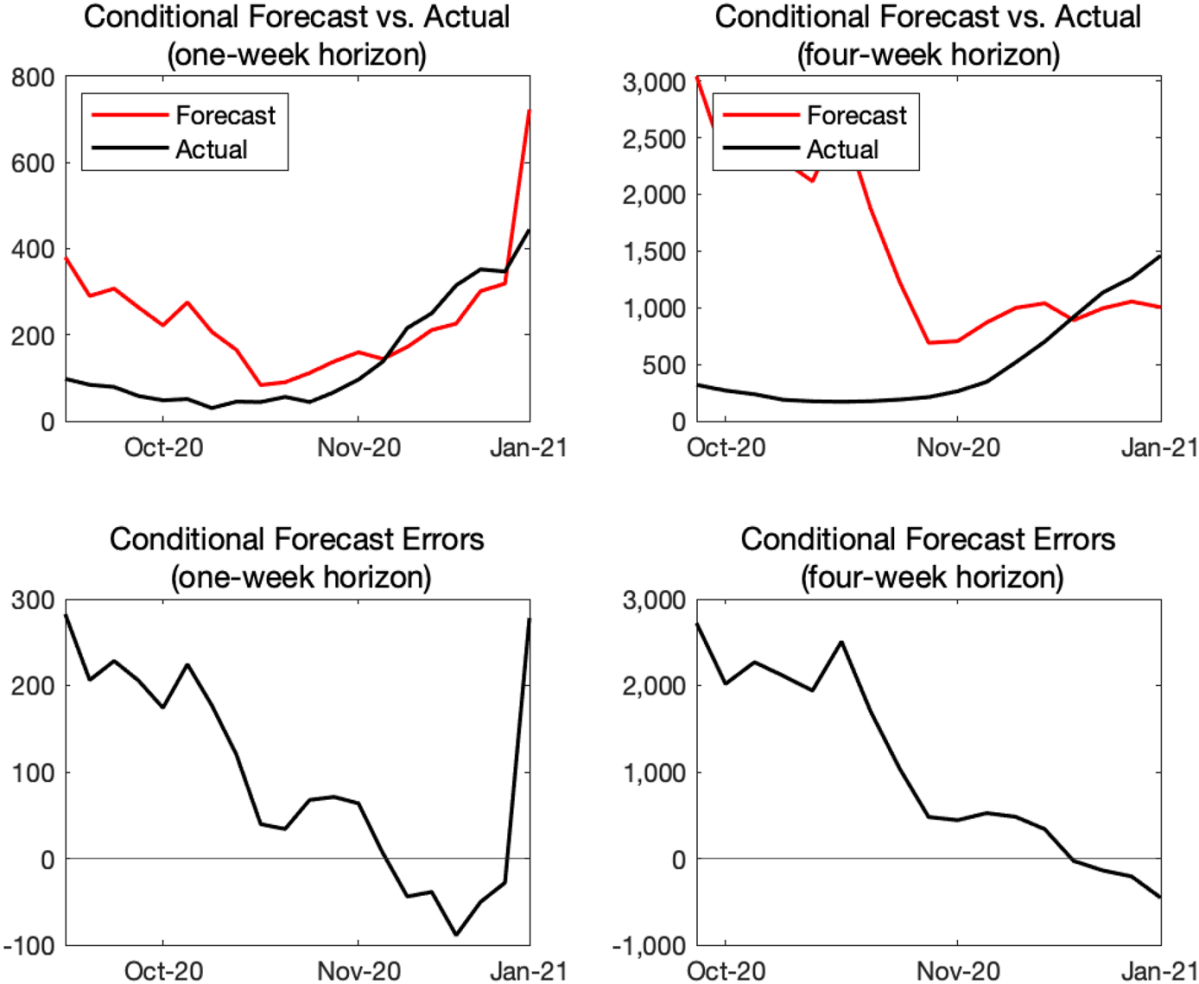
Real-time forecast evaluation: new deaths

Top panels in Fig. 11 show actual and forecasted outcomes for the number of new COVID-19 deaths for 1- and 4-week horizons, whereas bottom panels show their forecast errors. For both 1- and 4-week horizons, our model has over-predicted the new COVID-19 deaths most of the time. For the 4-week horizon, the forecast errors for October and November 2020 are sizable.

Since we use the average of $\beta _{t}$ in the past 5 months to generate future projections, we tend to over-estimate the new infections if the 5-month time window includes higher values of $\beta _{t}$. In October 2020, we use the average of $\beta _{t}$ from May to September, and the time window includes June and July in which a spike of $\beta _{t}$ is observed. Due to these higher values of $\beta _{t}$, the estimated $\beta$ for projection at the time of October 2020 is high leading to the over-prediction. This applies to the estimation of $\delta$ as well. The death rate was very high from May to July 2020, and hence, the conditional forecasts of deaths as of October 2020 (both 1-week-ahead and 4-week-ahead forecasts) are higher than the actual values.

We use the simple past averages of $\beta _{t}$ and $\delta _{t}$ for projection because our main purpose is to illustrate the relationship between economic loss and infection over 1-year horizon, not precisely forecasting the paths of new infections and deaths over 1- or 4-week horizons. As shown in the bottom-left panel of Fig. 3, the path of $\beta$ fluctuates and does not exhibit any predictable patterns. In this case, the past 5-month average is not a bad choice for the projection over next 12 months. If there is a factor which reasonably affects the future paths of $\beta$ or $\delta$, we will make parameter adjustments as shown in later sections.

## Policy analyses

### Criteria for lifting the SOE in Tokyo (January 2021)

Analysis in this subsection was conducted in January 2021.

The Japanese government declared the state of emergency in Tokyo and several other regions on January 7th, 2021, in an attempt to slow the spread of COVID-19. Since then, Governor of Tokyo has asked individuals to stay at home after 8 p.m., restaurants to close their doors by 8 p.m., and offices to reduce the number of workers going to the office by 70%, etc. Some officials have suggested that they would like to maintain the state of emergency until the number of new infections is down to around 500 per day in Tokyo. For the week ending in January 17th, 2021, the average number of new daily infections was 1504.

In this section, we use our model to examine the consequences of adopting alternative stopping criteria for the current state of emergency in Tokyo.^19^ To do so, we recalibrate our model using the numbers of new infections, new deaths, and the mobility index in Tokyo.

We also construct a measure of monthly output in Tokyo. To conduct our analyses, we need high frequency (at least monthly frequency) data on economic activity for each prefecture, but there is official statistics of the prefecture-level monthly output. Please see Fujii and Nakata (2021) for more details on how to construct a measure of monthly prefecture-level output in Japan using publicly available data. Here, we lay out the basic idea of how to construct such measures. Indices of Tertiary Industry Activity and Indices of Industrial Production, compiled by the Ministry of Economy, Trade and Industry (METI), report monthly output indices for many sectors in service and manufacturing industries. The Economic Census for Business Activity, published every 5 years, reports sectoral value added in each prefecture. Using the sectoral value-added shares in 2016 and assuming they are fixed over time, we compute the value-added weighted average of the monthly output indices, which is our measure of prefecture-level monthly gross domestic product (GDP). The Cabinet Office publishes Regional Domestic Expenditure Index (RDEI) in every quarter, which is a measure of prefecture-level monthly gross domestic expenditure (GDE). For those months in which these two measures, monthly GDP and GDE, are available, we take a simple average to compute our measure of prefecture-level monthly gross domestic output (GDO). For the most recent months in which some of those data are not available yet, we nowcast monthly GDO using mobility and data from Teikoku Databank (TDB) as predictors.

We consider three scenarios. In the first scenario, the economic activity during the state of emergency is such that the number of new daily cases reaches 500 in the eighth week. We refer to this scenario as the baseline scenario. In the second and third scenarios, the number of new daily cases reaches 500 in the fourth and twelfth week, respectively. We refer to these two scenarios as rapid- and gradual-decline scenarios. These scenarios are implemented by adjusting the level of $\alpha$ during the state of the emergency.

We assume that, once the emergency period ends, $\alpha$ declines to 4%, its average level from September 2020 to November 2020 according to the monthly GDP for Tokyo we constructed. We also assume that, if the number of new daily cases increases to 2000 after the end of the current emergency period, there will be another emergency declaration, which would be in place until the same stopping criterion is met. This assumption is motivated by our observation that the hospital capacity constraint seems to have become a pressing concern when the number of new infections approached this threshold in the recent past. It would be useful to examine the benefit of expanding the hospital capacity and increasing the threshold for the second emergency declaration, but such exercise is outside the scope of the paper.

Finally, we assume that the pace of vaccine distribution in Tokyo is such that $V_{t}$ increases from zero in the last week of March, 2021 to 160,000 in the final week of June, 2021.^20^

**Fig. 12:**
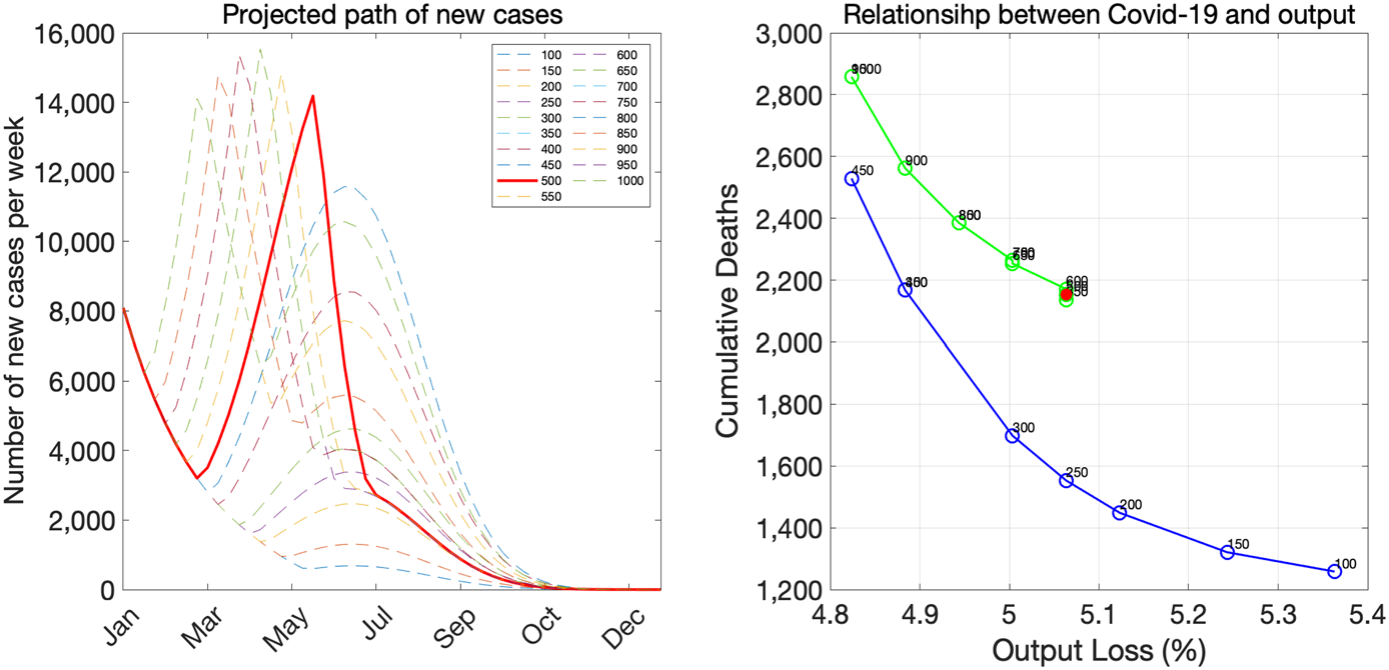
Baseline scenario

The left panel of Fig. 12 shows the paths of new infections under the baseline scenario with alternative criteria for ending the state of emergency. The solid red line shows the path when the stopping criterion is 500 per day (or 3500 per week), whereas other thin dashed lines shows the paths under alternative criteria. One important feature of this panel is that, if the stopping criterion is sufficiently high and the current emergency period is sufficiently short-lived, there will be a second emergency declaration down the road. Otherwise, vaccines will arrive on time to avoid the second emergency declaration. The stopping criterion of 500 is slightly above the threshold value: it leads to another emergency declaration in late May.

The right panel of Fig. 12 shows pairs of cumulative deaths and the output loss associated with various stopping criteria. Note that, when the stopping criterion leads to another emergency declaration, the pair of deaths and the output loss is interior to the trade-off frontier shown in the blue color. The light green curve located in the northeast of the frontier curve consists of pairs of deaths and the output loss under stopping criteria that eventually lead to the second emergency declaration. If the stopping criterion is 450 or below, then the economy avoids the second emergency declaration and the pair of deaths and the output loss is on the trade-off frontier. The virtue of avoiding the second emergency declaration in our exercise is reminiscent of the virtue of avoiding the second wave in standard SIR models. See Moll (2020) who elucidates how a loose lockdown that avoids the second wave can save more lives than a strict lockdown that eventually leads to the second wave.

**Fig. 13:**
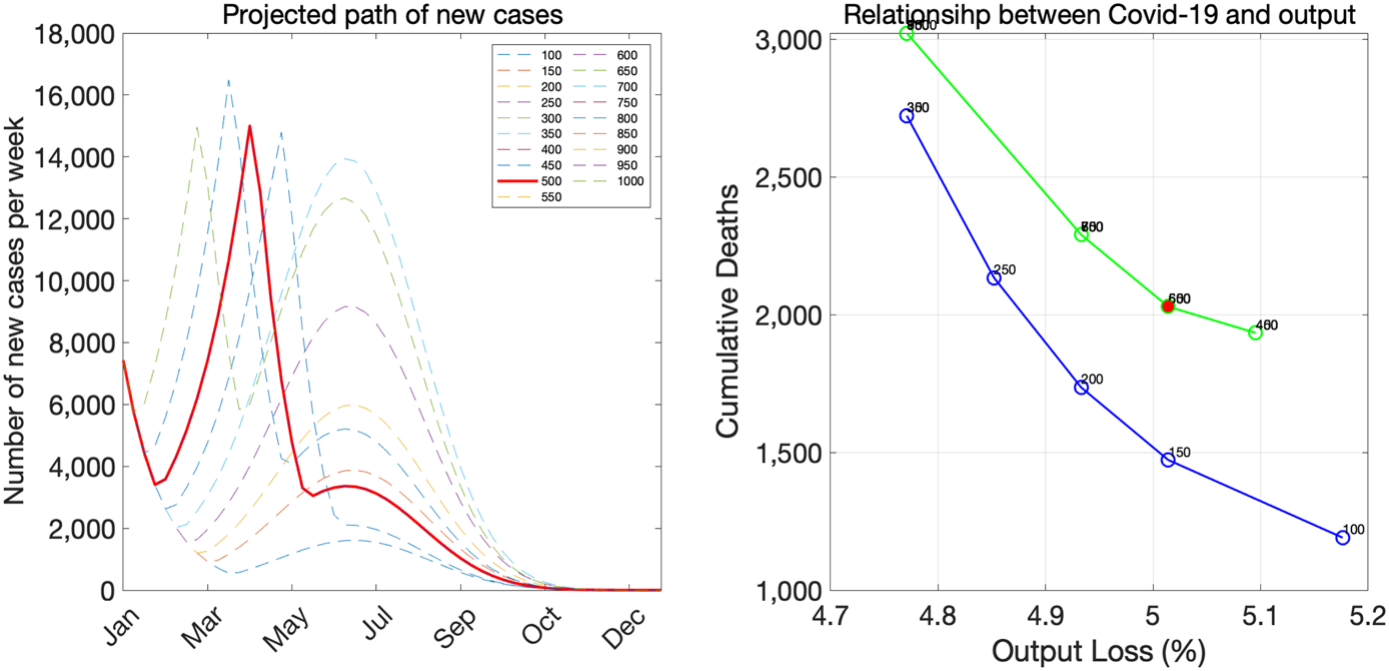
Rapid-decline scenario

Figure 13 shows the results for the rapid-decline scenario. In this scenario, the stopping criterion of 500 again leads to another emergency declaration later on, and as a result, the death-output outcome is inside the frontier curve. Avoiding another emergency declaration by setting the stopping criterion to, say 200, would improve both health and economics outcomes.

**Fig. 14:**
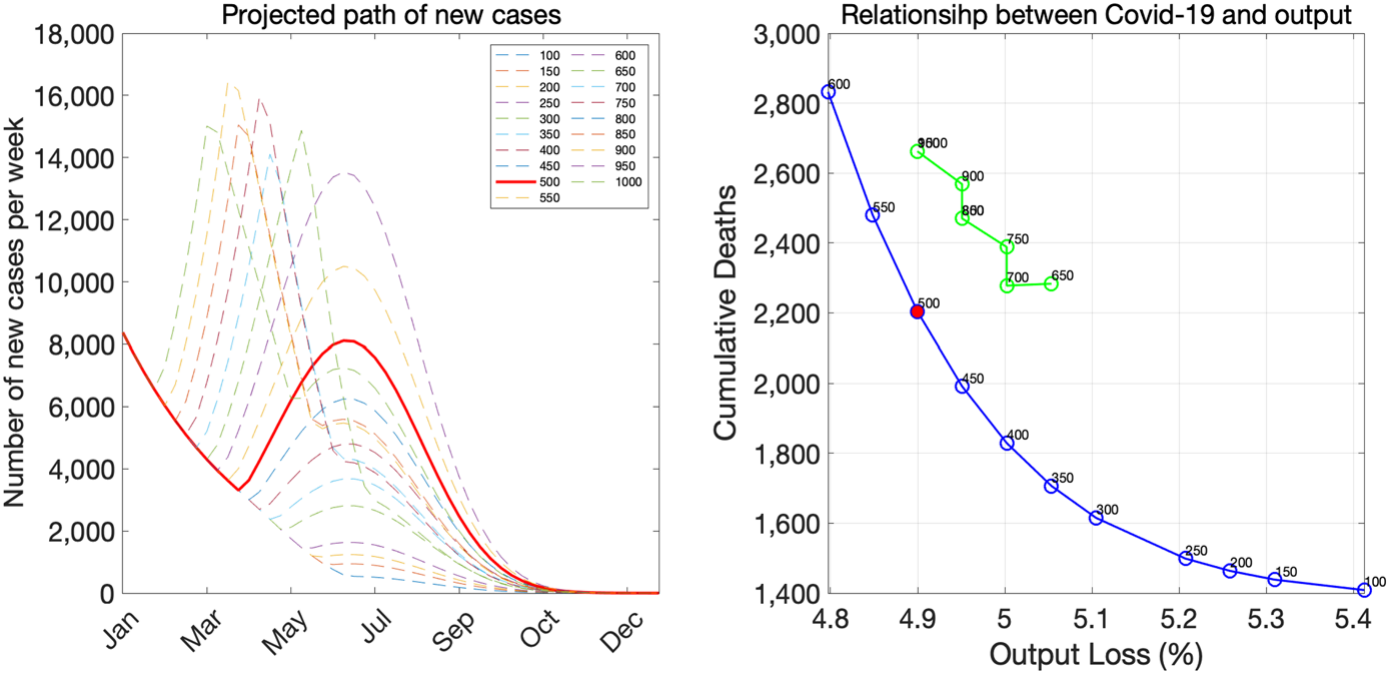
Gradual-decline scenario

Figure 14 shows the results for the gradual-decline scenario. In this scenario, the pair of death and the output loss associated with the stopping criterion of 500 is on the trade-off frontier because it does not lead to the second emergency declaration.

**Fig. 15:**
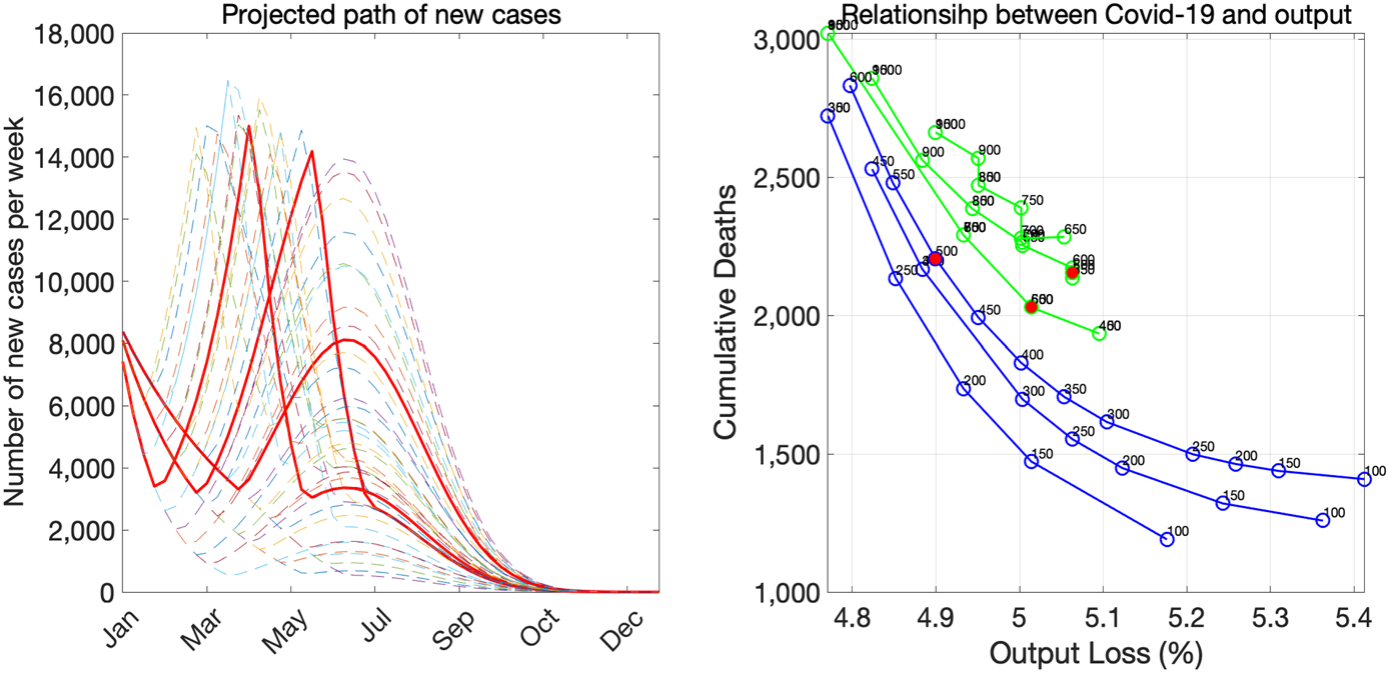
All scenarios

Figure 15 shows all three scenarios together. Several lessons emerge from the right panel. First, the first-best strategy is the strategy of a rapid decline with a low stopping criterion because the rapid-decline scenario is associated with the best trade-off frontier among all scenarios and a low stopping criterion allows the economy to be on the frontier curve. However, in countries like Japan in which the government lacks authorities to impose—or is reluctant to impose—strict social-distancing measures on its citizens, a rapid decline may not be possible. The next best strategy seems to be the strategy of a gradual decline with a moderate stopping criterion, which leads to pairs of deaths and the output loss that are in the middle part of the frontier curve.

There are two types of strategies that appear inferior to the aforementioned two strategies. The first is the strategy of a rapid decline with a high stopping criterion, which puts the economy at a high risk of inducing the second emergency declaration. The second is the strategy of a gradual decline with a low stopping criterion, which is associated with pairs of deaths and the output loss being in flat regions of the trade-off curve.

**Fig. 16:**
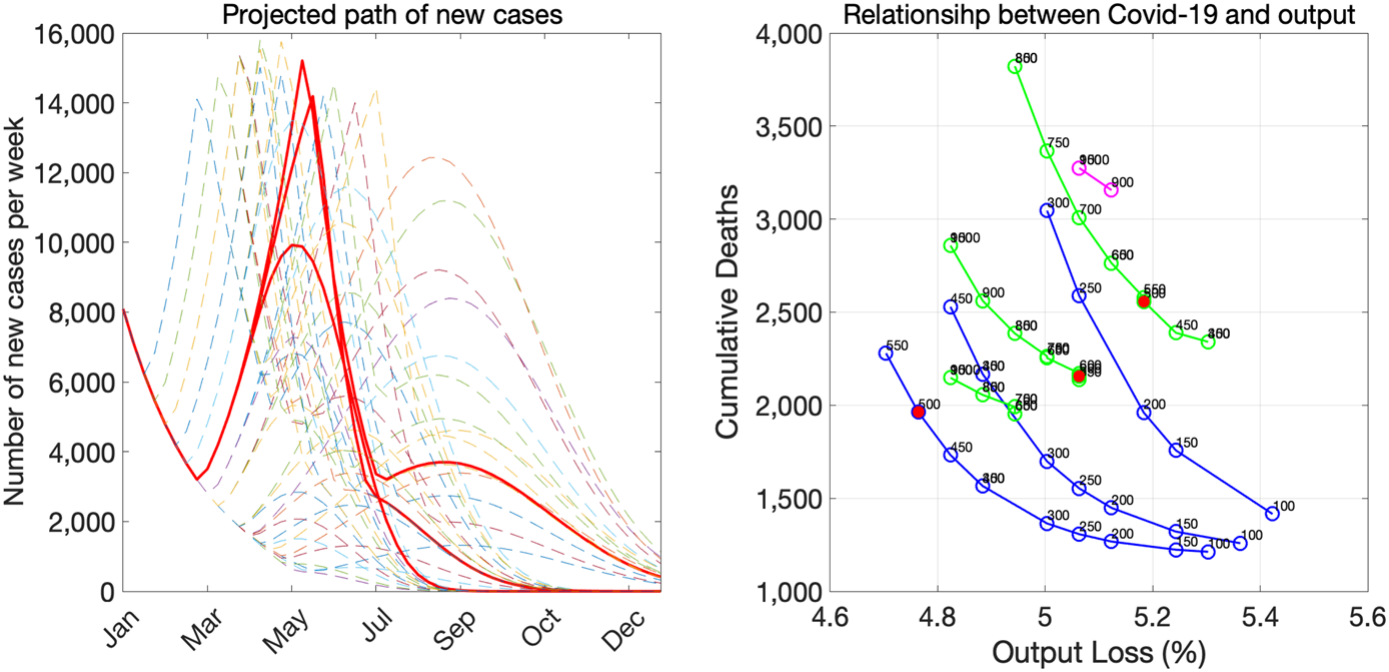
Alternative vaccine assumptions

We end this subsection by examining the implications of alternative vaccine assumptions for the effectiveness of alternative stopping criteria. Figure 16 shows the evolution of COVID-19 and the trade-off curves—shown in the left and right panel, respectively—under the baseline scenario with the baseline and two alternative vaccine assumptions. In the first alternative vaccine assumption, the value of $V_{t}$ is twice as large as that under the baseline at any *t*. In the second alternative vaccine assumption, the value of $V_{t}$ is half of that under the baseline at any *t*.

Let us first consider the first alternative vaccine assumption with the stopping criterion of 500. In this case, once the emergency status is over, the number of new cases increases and peaks in May at a level comfortably below the level that triggers the new round of the emergency period. Thus, the pair of deaths and the output loss is on the frontier curve, which is located on the southwest part of the frontier curve associated with the baseline vaccine assumption.

The frontier curve associated with the second alternative vaccine assumption is on the northeast part of the baseline frontier curve. Under this assumption and with the stopping criterion of 500, the government declares the second emergency declaration in early June. Thus, the pair of deaths and the output loss associated with the stopping criterion of 500 is interior to the frontier curve. To be on the frontier curve, the stopping criterion has to be 300 or less.

Figure 16 underscores how important it is to distribute vaccines at a faster pace. Provided that the government successfully avoids the second emergency declaration, the choice of the stopping criterion is about which point to choose on a given trade-off frontier. The optimal choice depends on various factors, including model specifications, assumptions, and one’s philosophy about life and death–factors about which empirical evidence may not be able to provide definite answers and two reasonable people can disagree with each other. In contrast, policies of distributing vaccines at a faster pace are desirable regardless of what these factors are, as better vaccine policies move the entire trade-off curve in the southwest direction in which both health and economic outcomes are better.^21^

### Spread of coronavirus variants (March 2021)

Analysis in this subsection was conducted in March 2021.

**Fig. 17:**
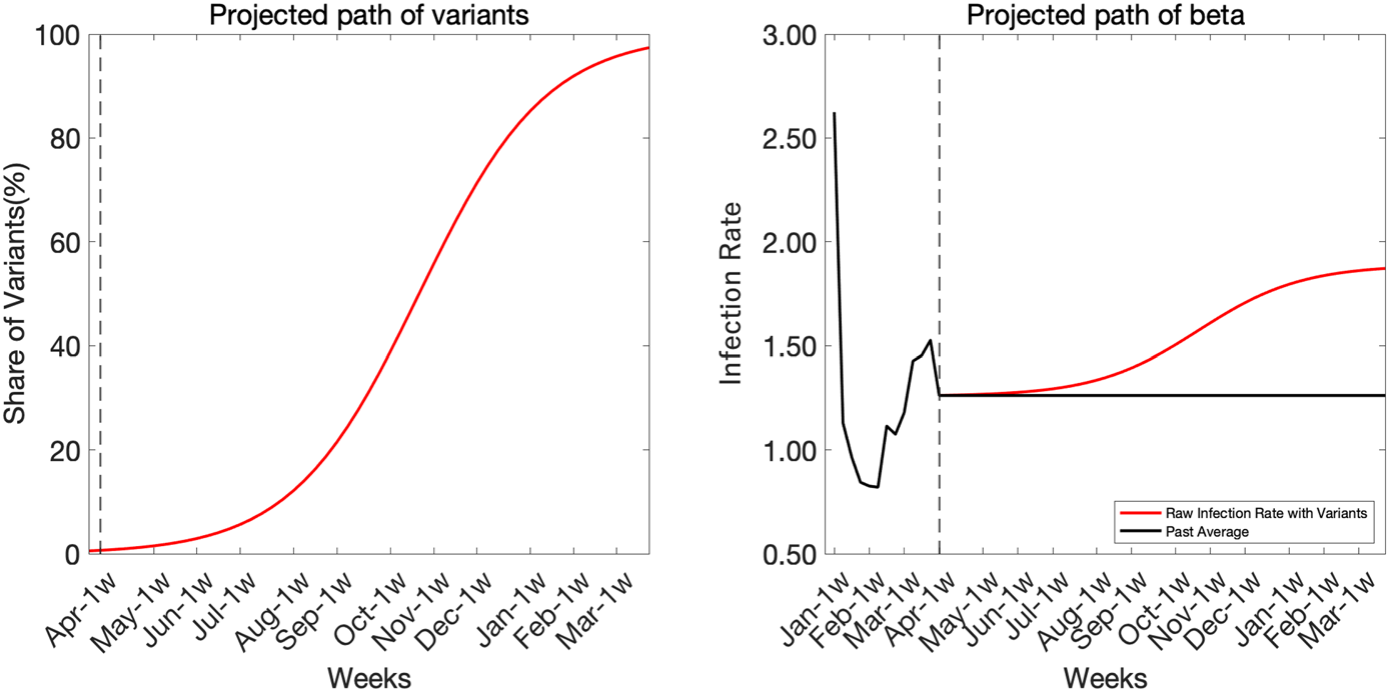
Spread of a more infectious variant

In March, several cases of the Alpha variant have been reported. This variant exhibits higher infection and mortality rate. One way to reflect the spread of the variants on $\beta _{t}$ is to use a logistic function. Let $p_{t}$ be the share of the Alpha variant in reported new cases. The share grows as follows

$$\begin{aligned} \ln \left( \frac{p_{t}}{1-p_{t}}\right) =\eta _{0}+\eta _{1}t, \end{aligned}$$

where *t* is the duration from the start of simulation, $\eta _{0}$ controls the variant share in the initial period, and $\eta _{1}$ controls the growth rate of the variant. Let $1+\kappa$ be the relative transmission rate of the Alpha variant to that of the then-prevalent variant (if it is 1.5 times more infectious, $\kappa =0.5$). Then, the path of transmission rate $\beta _{t}$ is given by the following equation

$$\begin{aligned} \beta _{t}=\left( 1+\kappa \right) p_{t}{\bar{\beta }}, \end{aligned}$$

where ${\bar{\beta }}$ is the past average of $\beta _{t}$. Theoretically, $\eta _{1}$ equals to $\kappa$, but we do not impose this restriction. Figure 17 displays the spread of variants and associated increase of $\beta _{t}$.

We consider several scenarios of spreading the Alpha variant and examine the risk to the economy and death toll. The fist scenario assumes slow spread of the variant, and the logistic growth parameter is set as $\eta _{1}=0.17$. The initial share of the variant is set as $p_{0}=0.55\%$. This scenario is consistent with the spread of the Alpha variant observed in the US. The second scenario considers fast spread of the variant by setting $\eta _{1}=0.52$ and $p_{0}=1.1\%$, which is consistent with the data in the UK. In both scenarios, we set $\kappa =0.5$.

**Fig. 18:**
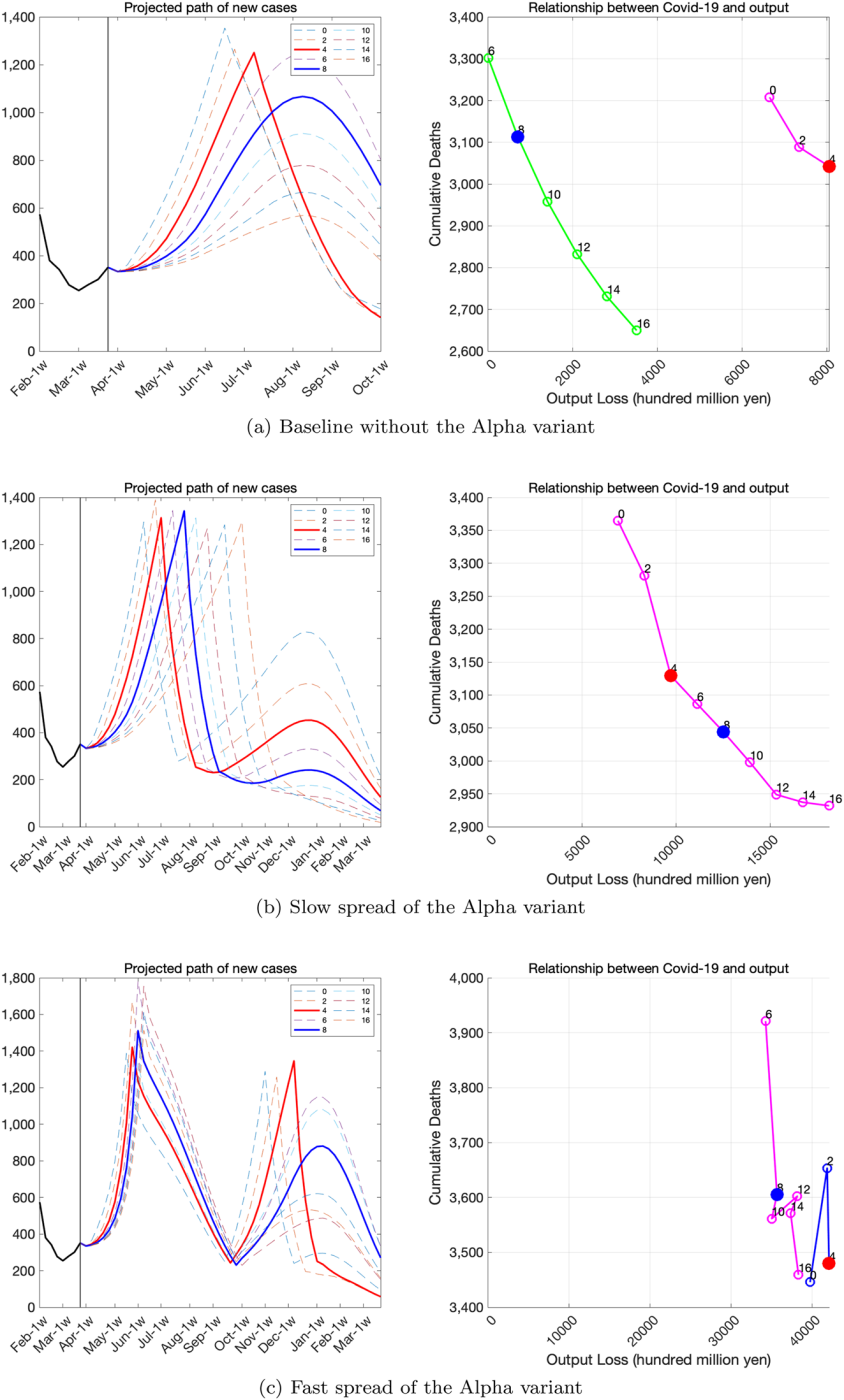
Risk of the spread of the Alpha variant

Figure 18 illustrates the risk of the spread of variants. In each panel, we show variations in the pace of economic recovery. The numbers in graph legends indicate the number of weeks it takes for the economy to recover from the level during the SOE to a higher level, which we set the level of autumn 2020. In the baseline case without variants (top panel), we can avoid another SOE if we recover the economy gradually. If the Alpha variant spreads even at a slower pace (middle panel), we cannot avoid another SOE and output loss will be much larger. If the speed of spread is faster (bottom panel), we may need to issue the SOE twice, which will exacerbate the economic damage even more.

### Faster vaccine distribution (June 2021)

Analysis in this subsection was conducted in June, 2021.

The third SOE was issued to Tokyo and other prefectures on April 26th, 2021 and announced to be lifted on June 20th. Compared to other major developed countries, Japan started vaccine rollout late, but acceleration has been remarkable. The number of vaccine shots reached a million per day for some days in June. In examining the effect of vaccine rollout pace in this subsection, we consider its effects not only on infection, but also on the number of severe cases, which we incorporated into our model in June. The laws of motion are given by the following equations

$$\begin{aligned} N_{t}&=\omega _{\beta ,t}\beta _{t}\frac{\left( 1-h\alpha _{t}\right) ^{2}}{POP_{0}}I_{t}S_{t},\\ S_{t+1}&=S_{t}-N_{t}-V_{t},\\ I_{t+1}&=I_{t}+N_{t}-\gamma I_{t}-\omega _{\delta ,t}\delta _{t}I_{t},\\ D_{t+1}&=D_{t}+\omega _{\delta ,t}\delta _{t}I_{t}. \end{aligned}$$

The number of severe patients at time *t* is given by^22^

$$\begin{aligned} H_{t+1}=H_{t}+\omega _{\delta ,t}\delta _{t}^{ICU}N_{t}-\gamma ^{ICU}H_{t}-\omega _{\delta ,t}\delta _{t}I_{t}, \end{aligned}$$

where $\delta _{t}^{ICU}$ is proportional to $\delta _{t}$, and $\gamma ^{ICU}$ is picked to match the past data best. The stock variable $H_{t}$ is constructed outside the SIRD model, and does not affect the dynamics of other variables. It is not another mutually exclusive compartment, so $S_{t}+I_{t}+R_{t}+D_{t}=POP_{0}$ still holds at any *t*. We take this modeling approach to keep the epidemiological part of the model as simple as possible while providing relevant projections of severe patients.

The difference between this subsection and Sect. 3 is the addition of parameter adjustment terms $\omega _{\beta ,t}$ and $\omega _{\delta ,t}$. There are two potential reasons for why the projected path of the transmission rate $\beta$ deviates from the actual path in the absence of judgmental parameter adjustment.

The first reason is that $\beta$ tends to be low during the SOE periods, while it is high outside the SOE periods. As a result, right after a SOE begins, the past 4-month average of $\beta$, which includes periods outside the SOE, tends to overstate the future path of $\beta$. Conversely, right after a SOE ended, the past 4-month average of $\beta$, which includes periods outside the SOE, tends to understate the future path of $\beta$.

The second reason is that our single-group SIR model, without appropriate parameter adjustment, can exaggerate the effect of the prioritized vaccine rollout to the elderly on the transmission rate for the reasons described in Sect. 6. In particular, when vaccines are distributed to elderlies first, a single-group SIR model over-predicts severe cases and deaths, while it under-predicts new infection.

For these reasons, it is convenient to allow for the possibility of adjustment on the future path of beta. To facilitate such adjustment, we introduce the following AR(1) adjustment term to the future path of beta

$$\begin{aligned} \omega _{\beta ,t+1}=1+\lambda \left( \omega _{\beta ,t}-1\right) . \end{aligned}$$

We pick $\lambda =0.95$ and $\omega _{\beta ,0}=1.1$ where $t=0$ is the start of projection. This adjustment amplifies the number of new cases, and its effect decays over time. We chose the AR(1) shock process because it is simple, yet flexible enough to respond to many situations. For example, we can set $\lambda =1$ or $\lambda >1$ if there is any reason to believe that the positive shock does not decay or amplify in the future.

As vaccines are distributed to the elderly, the share of older individuals whose mortality rate is much higher compared to the young, among the infected will decline. This will be further discussed in the next section. To reflect this composition effect, the mortality rate $\delta _{t}$ and the risk of severe symptoms $\delta _{t}^{ICU}$ will decline as the vaccine rollout for the elderly progresses. The adjustment term $\omega _{\delta ,t}$ is tied to the share of the elderly among the infected as shown in Sect. 6.2.

**Fig. 19:**
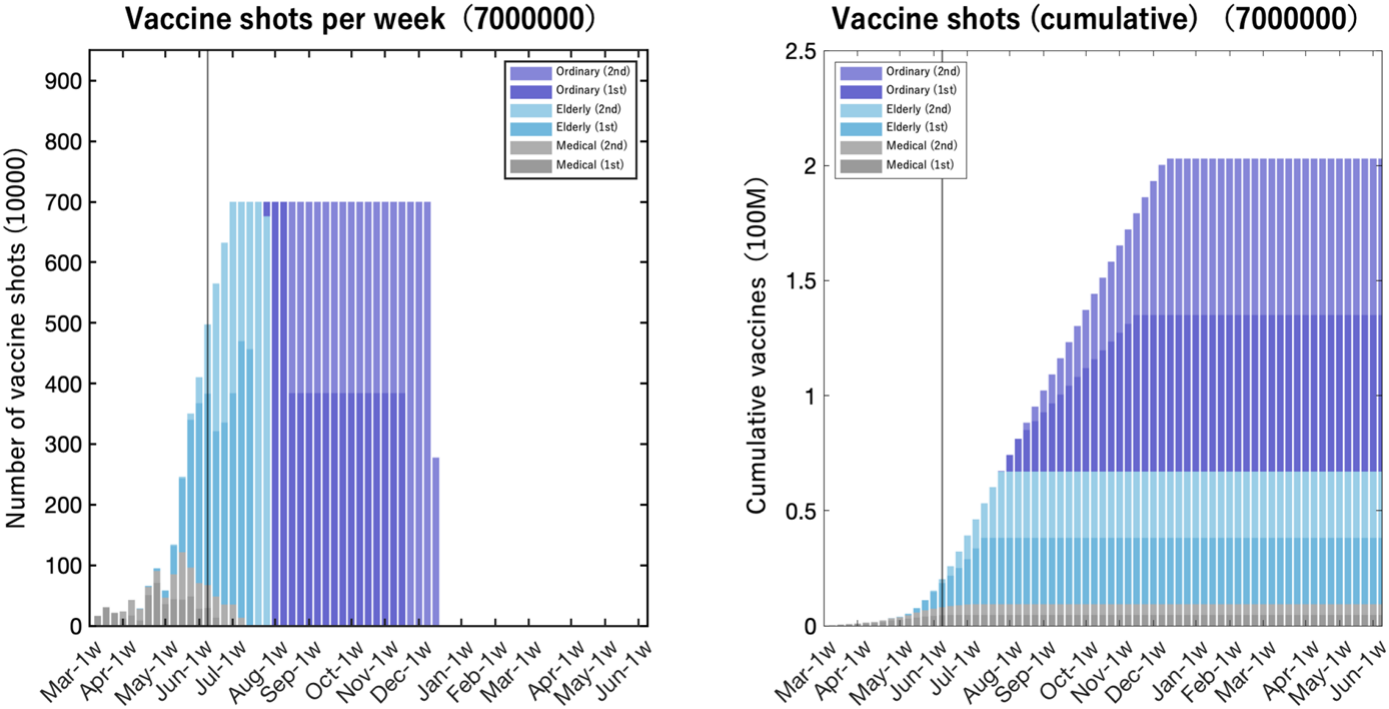
Vaccine rollout (1 M/day at the national level)

We explore the ramifications of various vaccine scenarios. The setup is as follows. Once the SOE in Tokyo is lifted in the fourth week of June, the economy will boost to the level of February in 2020 (right before the pandemic) within 12 weeks. We assume that the threshold of daily new cases to declare the SOE will increase to 1500 per day because of the declining mortality rate. Only 80% of population will be vaccinated. The efficacy of vaccines is the same as in Sect. 3: $E_{1}=0.625$ and $E_{2}=0.895$. We incorporate the spread of Delta variant as in the previous section. It is assumed to be 1.95 times more infectious, and will take over 80% share by the end of August. We consider various vaccine rollout paces from 0.6 to 1.3 M/day. The outlook of vaccine rollout of 1 M/day is depicted in Fig. 19.

**Fig. 20:**
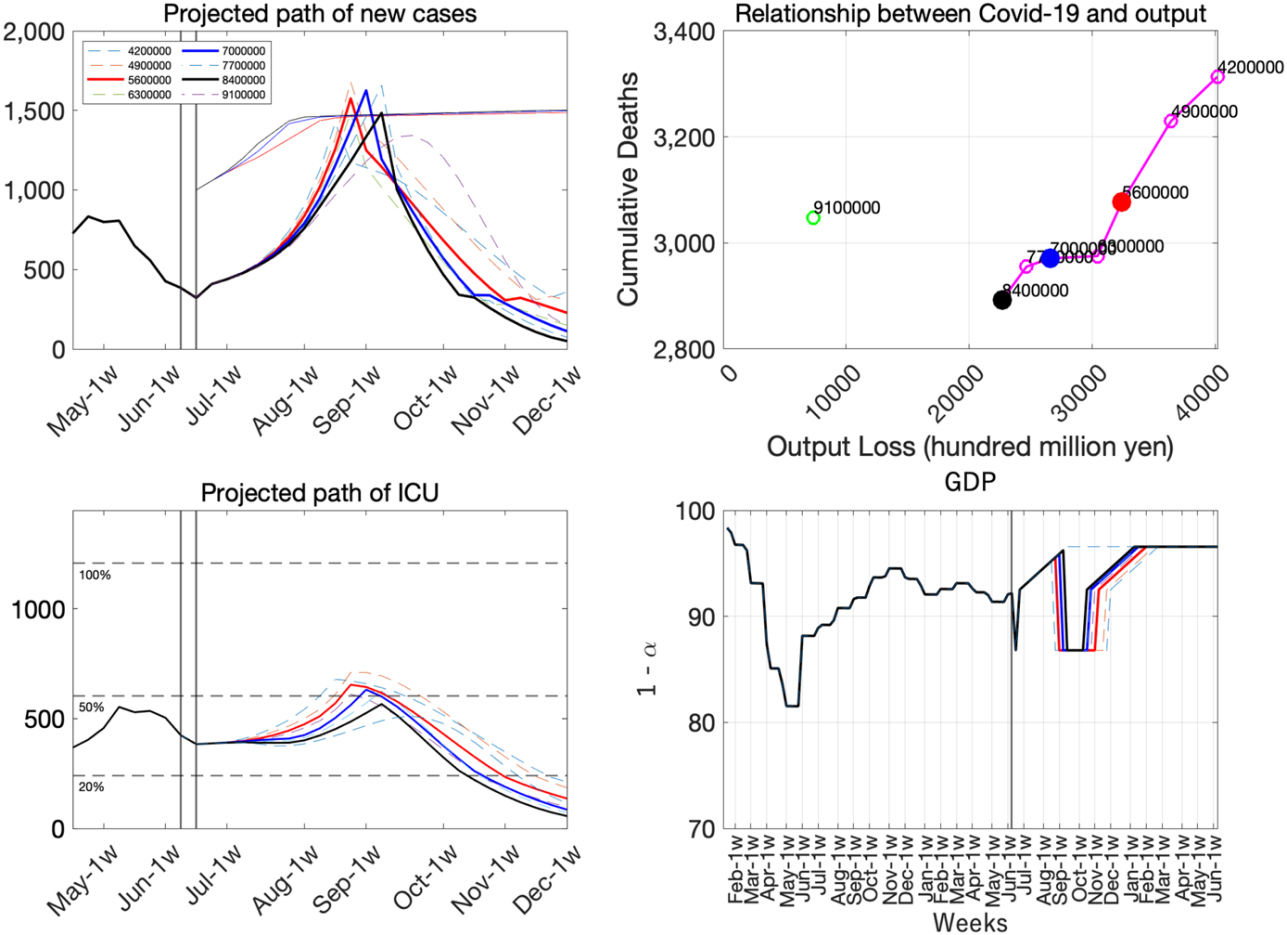
Various vaccine paces

The main results are summarized in Fig. 20. The top-left panel shows the projected path of new cases, bottom-left panel shows the projected path of seriously ill patients (ICU), the top-right panel shows the relationship between infection and economy, and the bottom-right panel shows the path of GDP. Under our assumptions, the next SOE is inevitable in almost all cases except the 1.3 M/day (9.1 M/week) case. From the top-right panel, we can see that faster pace of vaccine rollout reduces both output loss and deaths from infection. If we compare 0.6 M/day and 1.2 M/day, the difference in economic loss is 1.8 trillion yen and the that of cumulative deaths is around 400. If we can increase the rollout pace by 0.1 M/day, we can save dozens of people and hundreds of billions of yen.

## Age heterogeneity

Analysis in this section was conducted in June, 2021.

One of the key features of COVID-19 is its differential mortality risks across age groups. Older individuals exhibit much higher mortality than that of younger individuals. Table 3 shows mortality risk (the number of cumulative deaths/the number of cumulative PCR positive cases) by age group. The mortality risk of younger cohorts below age 40 is nearly zero while that of 70s is 4.8% and 80s+ is 13.2%. Also, contact patterns are different across age groups according to the social contact survey conducted by Munasinghe et al. (2019). Strong age-dependent assortativity is identified; individuals in the same age group interact more than different groups. This within-group contact rate is higher for younger people. The contact rate between the young and old is low implying that the benefit of vaccinating the old does not propagate to the young much.

As discussed earlier, we take these effects of age heterogeneity into account through parameter adjustments of the single-group SIR model. In this section, we explain the rationale of our approach.

We first compare the aggregate dynamics of a naive single-group model without parameter adjustment with those of a multi-group SIR model. We show that, when vaccines are distributed to the elderly before non-elderly, a naive single-group SIR model over-predicts severe cases and deaths, while it under-predicts new infection. We then show that an appropriate adjustment of parameters of the single-group SIR model generates aggregate dynamics that are identical to, or very similar to, those of the multi-group SIR model. We focus on the projections in Tokyo.

**Table 3:** Mortality risk by age group. Source:MHLW COVID-19 trends (Kokunai Hassei Doukou) as of May 19th, 2021

Age group	<10s	10s	20s	30s	40s	50s	60s	70s	80s<	Total
Cumulative PCR positives	21,655	49,334	150,642	101,020	98,371	89,181	57,428	51,057	50,616	669,304
Cumulative deaths	0	0	6	20	87	238	764	2441	6702	10,258
Mortality risk	0.0%	0.0%	0.0%	0.0%	0.1%	0.3%	1.3%	4.8%	13.2%	1.5%

### A multi-group SIR model

Individuals are partitioned into age groups indexed by $j=1,\ldots ,J$ with $P_{j}$ initial members. The total population is given by $POP_{0}=\sum _{j}P_{j}$. We consider a SIR compartment model for each group so that the following relationship holds at any given time *t*

$$\begin{aligned} S_{jt}+I_{jt}+R_{jt}+D_{jt}=P_{j}. \end{aligned}$$

The dynamics of the model is described by the following equations

$$\begin{aligned} N_{jt}&=\underbrace{K\left( 1-h_{j}\alpha _{t}\right) ^{2}\beta _{t}}_{{\tilde{\beta }}_{t}}\mu _{j}\frac{S_{jt}}{POP_{0}}\sum _{k=1}^{J}\rho _{jk}I_{kt},\\ S_{jt+1}&=S_{jt}-N_{jt}-V_{jt},\\ I_{jt+1}&=I_{jt}+N_{jt}-\gamma I_{jt}-\delta _{jt}I_{jt},\\ H_{jt+1}&=H_{jt}+\delta _{j}^{ICU}I_{jt}-\gamma ^{ICU}H_{jt}-\delta _{j}I_{jt}.\\ \end{aligned}$$

All variables are the same as in a single-group SIR model except a subscript *j*. The elasticity of economic loss on mobility $h_{j}$ can be heterogeneous across age groups but assumed to be common in this section for expositional purpose: $h_{j}=h$ for all *j*. A parameter $\mu _{j}$ denotes relative susceptibility of *j* to reflect differential risk of infection across age groups. Contact rates between groups *j* and *k* are captured by $\rho _{jk}$. By explicitly incorporating these contact rates, we can examine the effect of group-targeted policies such as prioritized vaccine rollout for the elderly more accurately.

We consider four age groups described in Table 4.

**Table 4:** Four age groups

Group	Age	Share (%)
$j=1$	0–19	16.7
$j=2$	20–39	21.3
$j=3$	40–59	27.6
$j=4$	60 +	34.4

The social contact matrix is borrowed from Munasinghe et al. (2019).^23^ The vector of relative susceptibility is chosen to minimize the error between the actual path of new infection in Tokyo and the simulated path given the contact matrix and mobility data. These parameters are set as follows

$$\begin{aligned} \mu =\left[ \begin{array}{c} 0.0491\\ 0.114\\ 0.0669\\ 0.0505 \end{array}\right] \text { }\rho =\left[ \begin{array}{cccc} 4.59 &{}\quad 0.97 &{}\quad 1.42 &{}\quad 0.45\\ 0.74 &{}\quad 2.30 &{}\quad 1.58 &{}\quad 0.91\\ 0.93 &{}\quad 1.35 &{}\quad 2.16 &{}\quad 1.18\\ 0.24 &{}\quad 0.63 &{}\quad 0.95 &{}\quad 2.10 \end{array}\right] \end{aligned}$$

The contact matrix $\rho$ shows active interactions among the same age group (higher values of diagonal elements). Also, we can see that the contact rate between the young and old is low. This fact implies that vaccine distribution to the elderly does not abate the spread of infection as much as in the single-group SIR model, where all agents are mixed equally.

We use the same $\alpha _{t}$ and $\beta _{t}$ as in the single-group SIR model. Other parameters are set as follows:

$$\begin{aligned} \delta =\left[ \begin{array}{c} 0.0001\\ 0.007\\ 0.172\\ 6.227 \end{array}\right] \times 0.01\times \frac{7}{16},\text { }\delta ^{ICU}=\left[ \begin{array}{c} 0.027\\ 0.054\\ 0.982\\ 8.698 \end{array}\right] \times 0.01\times \frac{7}{7} \end{aligned}$$

The 4-by-1 vector of mortality risks $\delta$ is computed by aggregating the numbers in Table 3. We assume that the average duration for an individual to stay in the state of *I* before going to *D* is 16 days. The age-specific risk of aggravation $\delta ^{ICU}$ is computed based on the report submitted to the MHLW-AB.^24^ The pace of vaccination is assumed to be 0.75 million per day (5.25 M/week) at the national level. Only 80% of population in all age groups will be vaccinated. Vaccines will be distributed to the age group of 60+ first, then 2/3 will be distributed to the group 40–59 and 1/3 will be distributed to the group 20–39. After that, age group of 0–19 will be vaccinated. At the start of simulation, the shares of each age group in the infected are 40%, 10%, 30%, and 20% respectively.

**Fig. 21:**
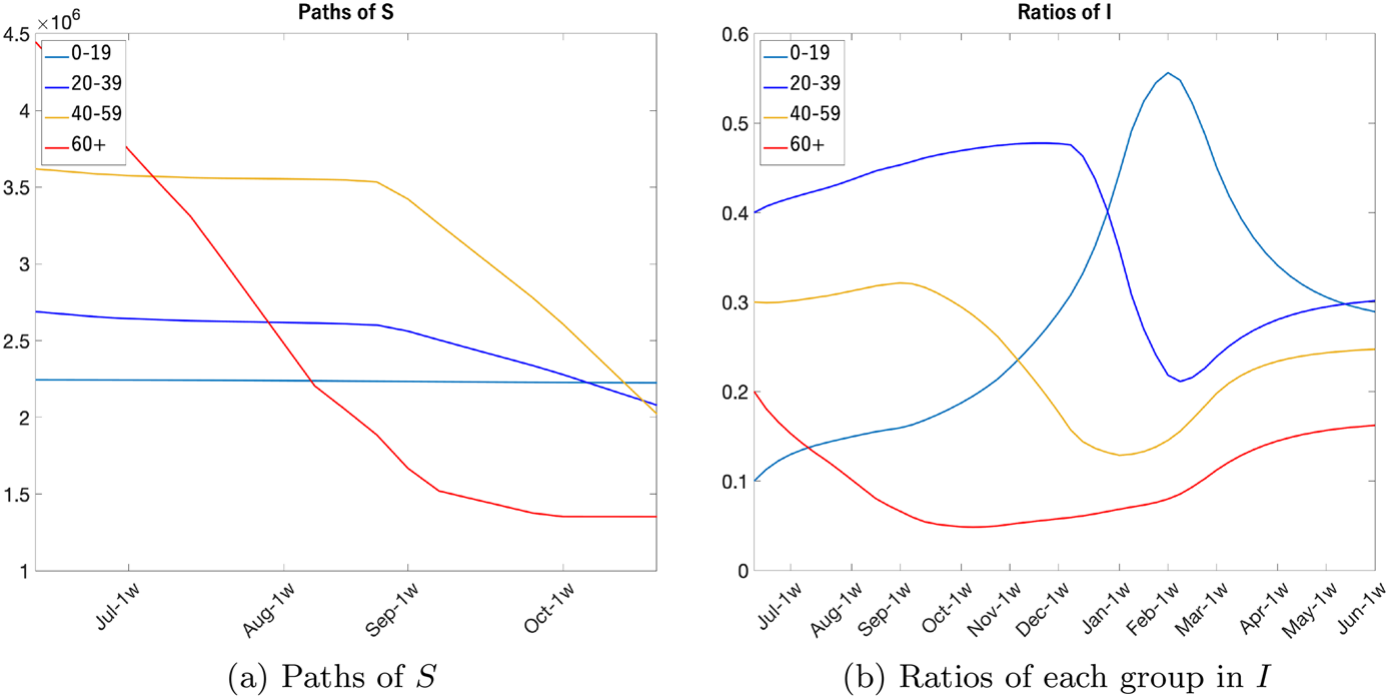
*S* and *I* in by age group

Figure 21 shows the projected paths of *S* and the shares of each group in *I*. Since the number of *I* is very small compared to the total population, the reduction in *S* mainly comes from vaccination. From the left panel of Fig. 21, we see the prioritized rollout for the group 60 +, then groups 40–59 and 20–39. Due to the vaccination, the share of group 60+ in *I* is decreasing up to October in the right panel of Fig. 21. The shares of other three groups are increasing. Since the group 40–59 is prioritized to 20–39, its share in *I* starts decreasing in September. Because of the vaccination of other three groups, the share of group 60+ will start increasing in October. Since the vaccination of group 0–19 is put off to the last, its share is increasing toward February of 2022, but once vaccination start, it will decrease. The shares of each group will eventually reach the steady state.

### Comparison between single-group and multi-group SIR models

Our baseline model described in Sect. 5.3 is a single-group SIR-macro model. In using this model, we employ two adjustments on time-varying parameters as discussed in Sect. 5.3; (i) an AR-1 type shock to $\beta _{t}$ and (ii) a declining path of $\delta _{t}$, reflecting the composition effect of the prioritized vaccine rollout. To understand the parameter adjustment we make, consider aggregating a multi-group SIR model as follows.

$$\begin{aligned} N_{t}&={\tilde{\beta }}_{t}S_{t}I_{t}\omega _{\beta ,t},\quad \text {where }\omega _{\beta ,t}=\sum _{j=1}^{J}\sum _{k=1}^{J}\mu _{j}\rho _{jk}\frac{I_{kt}}{I_{t}}\frac{S_{jt}}{S_{t}},\\ I_{t+1}&=I_{t}+N_{t}-\gamma I_{t}-\delta _{t}I_{t}\omega _{\delta ,t},\quad \text {where }\omega _{\delta ,t}=\frac{1}{\delta _{t}}\sum _{j=1}^{J}\frac{\delta _{jt}I_{jt}}{I_{t}},\\ H_{t+1}&=H_{t}+\delta _{t}^{ICU}I_{t}\omega _{\delta ,t}-\gamma ^{ICU}H_{t}-I_{t}\delta _{t}\omega _{\delta ,t}. \end{aligned}$$

For each aggregate variable, the functional form of the multi-group model is the same as that of the single-group model. The difference between them is that there are time-varying wedges ($\omega _{\beta ,t}$ and $\omega _{\delta ,t}$) that depend on the time-varying ratios of *I* and *S* in the multi-group model. Thus, if we compute these time-varying wedge parameters by first simulating from the multi-group model and feed them into the single-group model, the resulting aggregate dynamics of the “adjusted” single-group model are identical to those of the original multi-group model. Even without such perfect adjustment, a simpler computation of time-varying wedges allows the single-group model to generate aggregate dynamics that are quantitatively similar to those of the multi-group model. In what follows, we substantiate these two claims.

#### A naive single-group SIR model

Here, we compare the prediction of the multi-group model and a naive single-group SIR model. To compare the two models in a consistent manner, we need to pick an appropriate value of *K*. The constant should be

$$\begin{aligned} K=\frac{1}{\sum _{j}\sum _{k}p_{j}\mu _{j}\rho _{jk}q_{k}}, \end{aligned}$$

where $p_{j}$ is a population share of group *j* and $q_{k}=\frac{I_{k0}}{\sum _{j}I_{j0}}$ is the initial share of group *k* in *I*. With this value of *K*, the projection in the first period will be the same between the two models. Other parameters and settings are assumed to be the same as well except the two adjustments mentioned above.

**Fig. 22:**
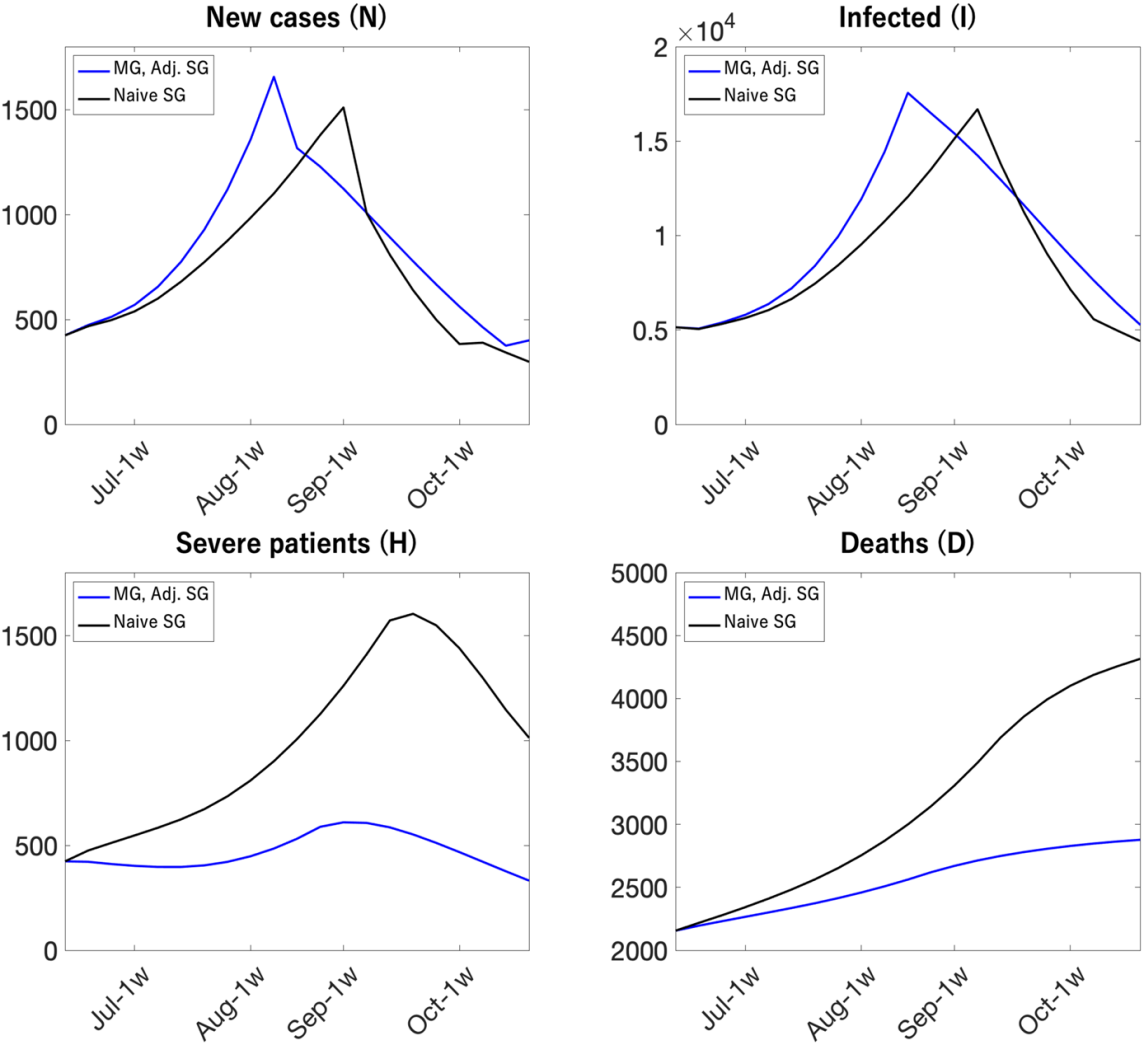
Multi-group SIR model, a naive single-group SIR model, an adjusted single-group SIR model (perfect adjustment)

Blue and black lines in Fig. 22 show the dynamics of the multi-group and naive single-group models, respectively. According to the figure, this naive single-group model over-predicts the path of deaths and severe cases and under-predicts the path of new infection compared to the multi-group model when vaccines rollout are more concentrated on the elderly.

#### Adjusted single-group SIR models

While a naive single-group SIR model cannot replicate the aggregate dynamics of the multi-group SIR model, parameter-adjusted single-group SIR model can.

**Fig. 23:**
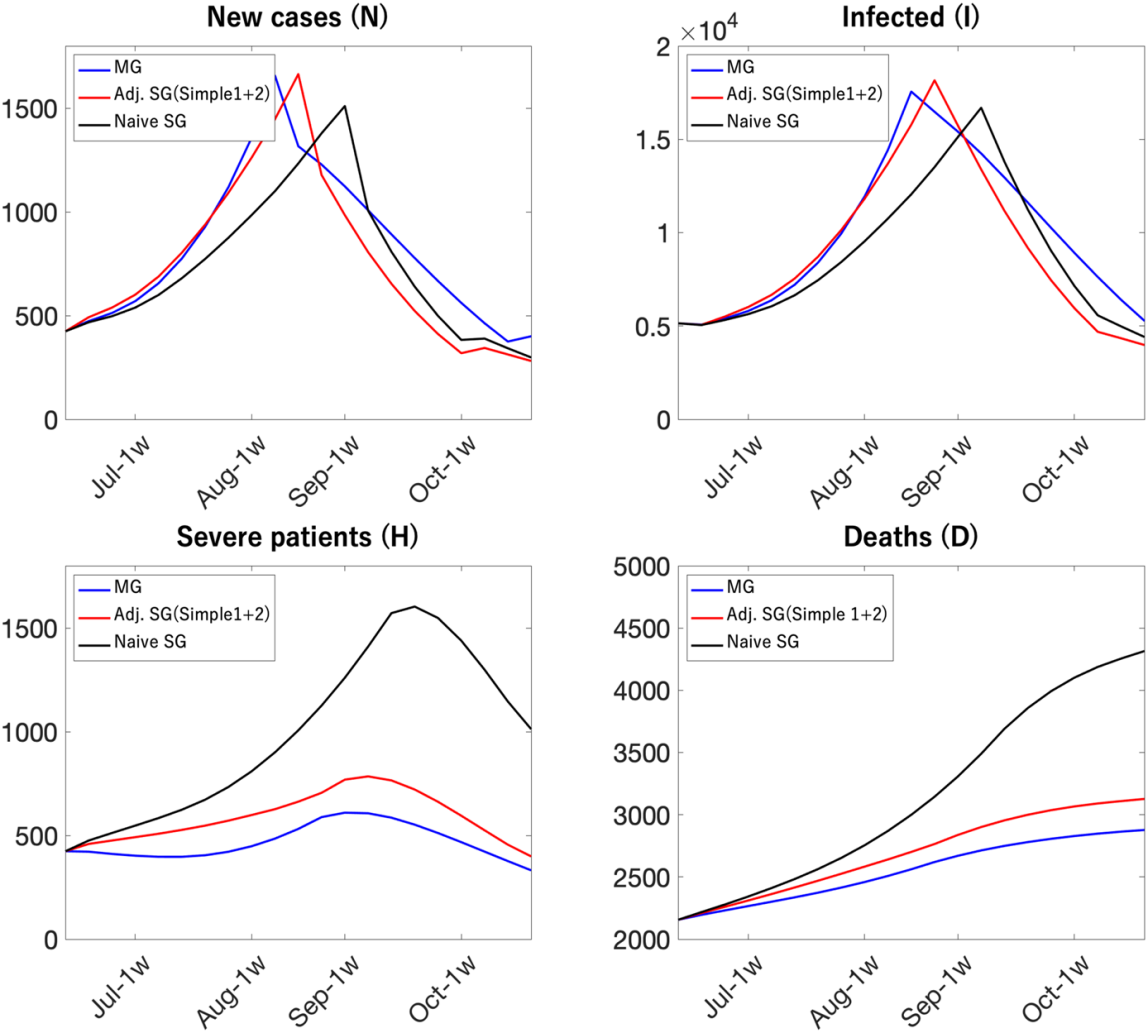
Multi-group SIR model, a naive single-group SIR model, an adjusted single-group SIR model (simple adjustment)

Blue lines in Fig. 22 show that the aggregate dynamics from the multi-group model coincides with those from the perfectly-adjusted SG SIR model where the simulation of the multi-group model was used to compute the sequence of the time-varying wedge. Figure 23 shows the aggregate dynamics from the multi-group model, the naive single-group model, and a single-group SIR model where we use simple procedures to capture the effects of the uneven distribution of vaccine rollout on the time-varying wedge. According to the figure, the single-group SIR model with a simple parameter adjustment delivers aggregate dynamics that are quantitatively similar to those of the multi-group model.

Although the single-group SIR model with simple parameter adjustments can generate similar aggregate dynamics of the multi-group SIR model, it is essential for researchers to use the multi-group model if the question of interest entails disaggregate dynamics themselves, or group-targeted policies as in Acemoglu et al. (2021). For instance, if estimates of the age-specific elasticity of economic loss on mobility ($h_{j}$) are available, one can investigate the effects of group-targeted lockdown policies or “vaccine passports”. Multi-group SIR models can provide more convincing analyses on these targeted policies than single-group SIR models.

In our weekly analysis, we use a single-group SIR model with simple parameter adjustment due to data limitation. In Japan, data on age-distribution of newly infected, severe cases, and deaths are not made publicly available in a timely manner. A single-group model with adjustments can buy both accuracy of projections and practicality for timely policy analyses.

## Conclusion

We build a tractable SIR-macro model with time-varying parameters and quantify the relationship between the spread of COVID-19 and output in Japan. We then used the framework to investigate various policy questions such as when to lift the state of emergency (SOE).

As discussed in Sect. 2, our model has contributed to the policy debate on how to control infection while minimizing the economic loss in Japan. This type of model can contribute to the policy debate in other countries as well. We hope that future research will lead to richer models that can better assist policymakers combat the next pandemic while minimizing social and economic costs of NPIs.

## Figures and Tables

**Fig. 24:**
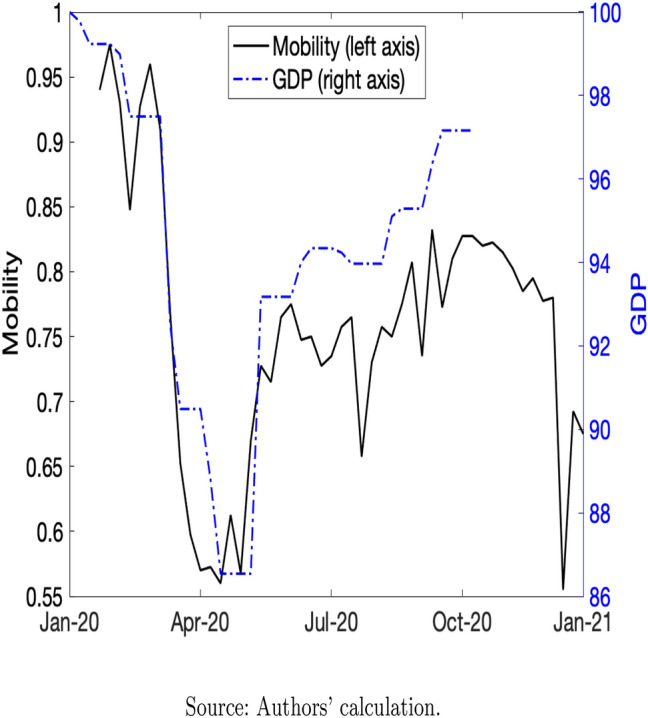
Monthly GDP in Tokyo. Source: Authors’ calculation

## Appendix 1: Constructing the reference level of output

This section was written in January 2021.

Our reference level of output at time *t* is the level of output at time *t* that would prevail if people did not impose any economic restraints at time *t* (that is, if people conducted their economic activities as if COVID-19 suddenly and magically disappeared at time t), taking as given the fact that the economy has suffered from the COVID-19 up to time t.

The reference level of output is different from the level of output consistent with pre-crisis trend (that is, the level of output that would have prevailed if there had been no COVID-19 crisis at all) because the reference level of output reflects the possibility that the COVID-19 crisis will leave permanent effects on the level of potential output. We would like our reference level of output to reflect the permanent effect of COVID-19 crisis on output because, otherwise, our $\alpha$ would be positive even at the steady state when people are not taking any social-distancing measures.

The reference level of output is different from the potential output because it reflects the cyclical position of the economy right before the COVID-19 crisis occurred. Suppose that the output gap was 5% right in the first quarter of 2020 and that output declines by 5% in the second quarter of 2020 due to social distancing measures. We would like to think that the 5% decline in output, and an associated decline in the mobility, reduced the transmission rate through $\alpha _{t}$ in this example.

We construct the reference level of output in two steps.

(i) In the first step, we construct the path of potential output. We apply the Bank of Japan’s estimate of the output gap in the fourth quarter of 2019 (1.2%) to GDP in December 2019 to construct potential GDP in December 2019.^25^ For January, February, and March of 2020, we apply the expected growth rate of potential GDP computed before the COVID-19 crisis by OECD (0.63%, annualized) to construct the potential output for these 3 months.^26^

For the remaining 9 months of 2020, we let potential GDP grow at a rate of 0.21% (annualized) so that the average growth rate of potential GDP in 2020 is 0.35%, consistent with the most recent OECD estimate.^27^ For 2021 and 2022, we let potential GDP grow at a rate of 0.24% and 0.21%, respectively, taking on board the December-2020 projection of OECD.

Let us provide some background information on why we used two sources, the Bank of Japan and OECD, in constructing the path of potential GDP. The estimates of the output gap from these two institutions for the past 3 years are very similar. In particular, their estimates of the output gap for 2019 are both 1.6%. We apply the fourth-quarter estimate of the Bank of Japan to our monthly GDP for December 2020 to compute the starting point of our monthly potential output. We rely on the BOJ’s quarterly estimate because OECD computes annual estimates only. As the fourth quarter output declined nontrivially in the fourth quarter of 2019 due to the consumption tax hike at the end of September 2019, using the fourth-quarter estimate of the output gap from the BOJ is more sensible than using the average estimate of the output gap for 2019 from OECD in constructing potential GDP in December 2019. We primarily rely on OECD in constructing the path of potential GDP from 2020 to 2022 because the Bank of Japan does not provide the growth rate of potential output.

(ii) In the second step, we add the expected cyclical component of output to the potential GDP path calculated in the first step. We take the level of the output gap in January 2020 as given and assume that the output gap will stay at that level throughout our simulation horizon.

All in all, our reference level of output grows very slowly over time and is essentially flat throughout our projection horizon.

## Appendix 2: Monthly GDP in Tokyo

This section was written in January 2021.

Figure 24 shows the Google mobility index and our monthly estimate of GDP in Tokyo. The figure looks qualitatively similar to the one for Japan as a whole shown in Fig. 2. Both mobility and output declined sharply in April 2020 and recovered gradually thereafter. The magnitude of the decline in the mobility was much larger in Tokyo than in Japan as a whole, likely reflecting the high population density of Tokyo which makes the mobility reduction imperative for the task of reducing mobility to contain the spread of COVID-19. The magnitude of the output decline in Tokyo was about the same as that in Japan, and the economic recovery in the fall of 2020 in Tokyo is not as strong as that in Japan.

## Footnotes

1 To fill in the gap, the Cabinet Office has funded teams of mathematical modelers from other disciplines and has asked them to provide the future outlook of infection and quantify the effects of various policies—from the effects of vaccine rollout to the consequences of holding the Tokyo Olympics. We joined this group in July 2021.

2 It was mid-June when several other researchers released their analyses of the Tokyo Olympics. See the 39th MHLW-AB meeting materials (held on June 16th, 2021) and simulation results organized by the Cabinet Office.

3 See the materials of Tokyo COVID-19 monitoring meeting.

4 See the slide “The risk of Delta variants”. It was mid-June when other researchers began to generate the outlook that incorporates the effect of the Delta variant. See the 39th MHLW-AB meeting materials (held on June 16th, 2021) and COVID-19 AI and Simulation Project by Cabinet Secretariat.

5 See Atkeson (2020) and Moll (2020) for the exposition of SIR models.

6 See Alvarez et al. (2020) and Farboodi et al. (2020) for similar matching functions.

7 Our use of a reduced-form model is similar in spirit to the widespread use of semi-structural models for policy analysis in central banks. For example, the FRB/US model—a semi-structural model of the US economy—is used prominently for the analysis of forward guidance policies at the Federal Reserve, whereas DSGE models—which suffer from the so-called forward guidance puzzle are not as frequently used as the FRB/US model. See Chung (2015) and Chung et al. (2018).

8 The monthly real GDP from the Japan Center of Economic Research can be accessed here: https://www.jcer.or.jp/en/economic-forecast/monthly-gdp. We assume the same GDP values for all weeks in the same month. If a week spans over two different months, its weekly GDP is prorated using two values.

9 SPI-M-O stands for the Scientific Pandemic Influenza Group on Modeling, Operational sub-group. The report “SPI-M-O: Summary of further modeling of easing restrictions – Roadmap Step 2” is available here.

10 See Fernández-Villaverde and Jones (2020) for a similar identification strategy.

11 Google’s COVID-19 Community Mobility Reports provide mobility indices across different categories of places at a daily frequency. We pick the following four categories as we think they compose a good measure of mobility which affects the overall transmission rate: parks, transit stations, retail and recreation, and workplaces. For each week, we compute the average of the median values of those four series to construct $M_{t}$. We use median values to eliminate the irregularity of holidays.

12 Our starting-date assumption is consistent with the statement made by Prime Minister Suga during a press conference in early January, 2021.

13 The most recent projections by OECD, IMF, and Word Bank are published in October 2020, December 2020, and January 2020, respectively.

14 Here, we are essentially saying that the raw transmission rate and the death rate are randomly distributed with population means and that we estimate their population means by the sample averages over the past 5 months.

15 Note that, in constructing the confidence set, we only consider parameter uncertainty—uncertainty that we have ways of quantifying, albeit imperfect. There is also uncertainty about the pace of vaccine distribution. Model misspecification generates another dimension of uncertainty. Thus, our confidence set should be seen as understating the true degree of uncertainty we currently face.

16 See Hall et al. (2020) for discussion of the value of statistical life in the context of COVID-19.

17 Source: https://www.npa.go.jp/publications/statistics/safetylife/jisatsu.html.

18 For readers unfamiliar with the idea of real-time forecast evaluation, see Faust and Wright (2009) and Clark (2011) for examples of real-time forecast evaluation exercises for macroeconomic variables.

19 The results shown in this section were generated on January 20th, 2021, using the data through January 17th, 2021.

20 160,000 per week in Tokyo is—in a population-adjusted term—is broadly in line with, 1.6 M per week in Japan assumed in the previous few sections because population in Tokyo is roughly 10% of total population in Japan.

21 The same goes to policies of reducing $\beta$ and increasing *h*. As we saw in the sensitivity analysis in Sect. 4.1.2, the economy can attain a lower number of deaths for any given output loss with lower $\beta$ and higher *h*.

22 When we introduced the severe cases into the model, we let the inflow into *H* depend on *N*. Early July, we modified the model so that the inflow depends on $I_{t}$ to be consistent with the literature.

$$\begin{aligned} H_{t+1}=H_{t}+\delta _{t}^{ICU}I_{t}-\gamma ^{ICU}H_{t}-\delta _{t}I_{t}. \end{aligned}$$

23 Munasinghe et al. (2019) provide a more disaggregated social contact matrix by conducting an internet-based questionnaire survey in Japan. The dataset covers all 47 prefectures and include a total of 1476 households. We aggregate their matrix to 4-by-4 using group population as weights.

24 See page 33 of the slide “National and prefectural epidemic curves” which was submitted to the AB meeting held on October 22nd, 2020.
